# Design and Synthesis of Coumarin Derivatives as Cytotoxic Agents through PI3K/AKT Signaling Pathway Inhibition in HL60 and HepG2 Cancer Cells

**DOI:** 10.3390/molecules27196709

**Published:** 2022-10-09

**Authors:** Safaa M. Kishk, Enas E. Eltamany, Mohamed S. Nafie, Roaa M. Khinkar, Rawan H. Hareeri, Sameh S. Elhady, Asmaa S. A. Yassen

**Affiliations:** 1 Department of Pharmaceutical Medicinal Chemistry, Faculty of Pharmacy, Suez Canal University, Ismailia 41522, Egypt; 2 Department of Pharmacognosy, Faculty of Pharmacy, Suez Canal University, Ismailia 41522, Egypt; 3 Department of Chemistry, Faculty of Science, Suez Canal University, Ismailia 41522, Egypt; 4 Department of Pharmacy Practice, Faculty of Pharmacy, King Abdulaziz University, Jeddah 21589, Saudi Arabia; 5 Department of Pharmacology and Toxicology, Faculty of Pharmacy, King Abdulaziz University, Jeddah 21589, Saudi Arabia; 6 Department of Natural Products, Faculty of Pharmacy, King Abdulaziz University, Jeddah 21589, Saudi Arabia; 7 Department of Pharmaceutical Organic Chemistry, Faculty of Pharmacy, Suez Canal University, Ismailia 41522, Egypt

**Keywords:** coumarin derivatives, MTT assay, apoptosis, PI3K/AKT, docking, drug discovery, industrial development

## Abstract

In this study, a series of coumarin derivatives, either alone or as hybrids with cinnamic acid, were synthesized and evaluated for their cytotoxicity against a panel of cancer cells using the MTT assay. Then, the most active compounds were inspected for their mechanism of cytotoxicity by cell-cycle analysis, RT-PCR, DNA fragmentation, and Western blotting techniques. Cytotoxic results showed that compound (**4**) had a significant cytotoxic effect against HL60 cells (IC_50_ = 8.09 µM), while compound (**8b**) had a noticeable activity against HepG2 cells (IC_50_ = 13.14 µM). Compounds (**4**) and (**8b**) mediated their cytotoxicity via PI3K/AKT pathway inhibition. These results were assured by molecular docking studies. These results support further exploratory research focusing on the therapeutic activity of coumarin derivatives as cytotoxic agents.

## 1. Introduction

Cancer is the world’s most important cause of premature death from non-communicable diseases [1]. Cancer is marked as an uncontrolled growth of abnormal cells due to dysfunction in apoptotic machinery [2,3,4]. Acute myeloid leukemia (AML) is the most common acute leukemia in adults and the second most popular type of leukemia in children. AML is distinguished by its poor prognosis and overall long-term survival for patients, despite current advances in cancer treatment [5]. The limited success of AML treatments, particularly in elderly patients, originates from the high toxicity of chemotherapeutic agents, as well as the heterogeneity of the disease [6]. On the other hand, Hepatocellular carcinoma (HCC) is an aggressive tumor that is the predominant primary liver malignancy. HCC is the second leading cause of cancer-related mortality and is characterized by its poor prognosis and high resistance to conventional chemotherapies [7]. According to WHO, liver cancer is the most common malignancy among Egyptians, with incidence and mortality rates of 19.7% and 29.4%, respectively [8].

Most chemotherapeutic drugs stimulated apoptotic cell death via the regulation of Bcl-2 family proteins. These proteins play an essential role in activating caspases and, hence, apoptosis induction [9,10]. Protein kinases play an important role in oncogenesis and are recognized as promising targets as antineoplastic agents. Numerous kinase inhibitors have been approved for clinical practice [11]. The PI3K/AKT signaling pathway is of vital importance in cell survival and growth [12]. PI3K/AKT pathway can regulate the activity of Bcl-2 family members and, consequently, is linked to the regulation of intrinsic apoptosis [13]. Furthermore, PI3K/AKT pathway inhibition can lead to cell-cycle arrest [14]. Therefore, PI3K/AKT pathway inhibition is a luminous target for treating human cancer [15].

Natural products are an imminent rich source of drug leads with diverse bioactivities, including anti-cancer agents [16]. For example, TIR-199, an immunoproteasome inhibitor of the syrbactin-class obtained from the bacterium *Pseudomonas syringae pv syringae* exhibited potant cytotoxicity in multiple myeloma, triple-negative breast cancer, (TNBC) and non-small cell lung cancer lines. Moreover, in a mouse xenograft model, small doses of TIR-199 displayed in vivo antitumor activity by attenuating myeloma-mediated bone degeneration [17].

Natural and synthetic coumarins have attracted intense interest in research as innovative and selective anti-cancer agents [18]. The 2*H*-chromen-2-one (coumarin) was first isolated in 1820 by Vogel from Tonka beans [19]. Coumarins are present in considerable amounts in cinnamon and several edible plants [20,21] and widely distributed in two plant families: Umbelliferae (Apiaceae) and Rutaceae [22]. Coumarins exhibit a diverse range of pharmacological activities such as anti-HIV [23], antitumor [24], antihyperlipidemic [25], and antihypertensive [26]. Their diverse pharmacological activities brought this class of natural products to the forefront. The design of coumarin skeleton substitutions has a major influence on their therapeutic applications [27].

Coumarins exert their anti-cancer activity through different mechanisms, either by telomerase and protein kinase inhibition [28,29], oncogene expression down-regulation [29], or by apoptosis-induction via activating caspase 9 levels. Researchers have also shown that coumarins can suppress the proliferation of cancer cells by arresting cell cycles at G0/G1 [28] and G2/M phases [30] and through P-gp inhibitors in cancer cells [31]. Hydroxycoumarins have also been reported to perform their anti-cancer activities by generating free radical species in cancer cells that generate oxidative stress and trigger a pro-apoptotic effect [32]. The *δ*-lactone coumarin ring has been demonstrated to be of fundamental importance for both the production and stabilization of these species and for the pro-apoptotic action of hydroxycoumarins [32]. Furthermore, 7-hydroxycoumarin derivative antiproliferative activity could be due to its effect on cancer cells with mitochondrial thiol compounds [31].

**Figure 1:**
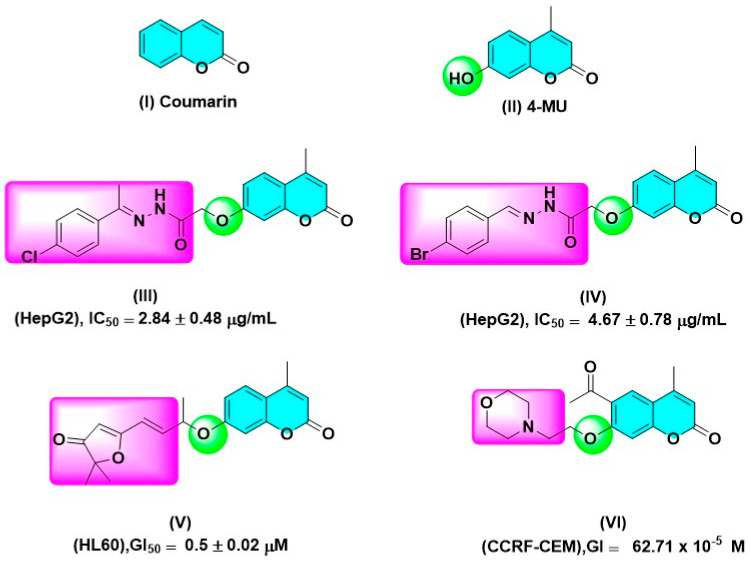
Selected structures of some anti-cancer coumarin-based derivatives.

7-Hydroxy-4-methylcoumarin (4-methylumbelliferone, 4-MU) is a naturally occurring antitumor coumarin obtained from several Apiaceae plants [33] and reported in *Cassia cinnamon* [34]. 4-MU is known to inhibit the synthesis of hyaluronic acid, which promotes tumor growth and progression [35]. Therefore, 4-MU acts as an effective chemo-preventive and therapeutic agent for pancreas, renal cell, prostate, ovarian, and breast cancers [20,36]. Thus, the *O*-substituted derivatives of 7-hydrox-4-methylcoumarins have attracted great attention in recent years. For example, the Prateeptongkum team has synthesized a series of combined 7-hydroxy-4-methylcoumarin **II** with different aryl hydrazide–hydrazones and screened them for their anti-cancer activity. Based on their results, compounds **III** and **IV** showed potent cytotoxic activity against HepG2 with IC_50_ values of 2.84 ± 0.48 µg/mL and 4.67 ± 0.78 µg/mL, respectively, compared to the standard doxorubicin (IC_50_ = 2.11 ± 0.13 µg/mL) [37]. The Viola group reported the synthesis of some modified analogs of geiparvarin (natural coumarin) and their biological assay against several human tumor cell lines. The new derivative **V** strongly induced apoptotic cell death in a promyelocytic leukemia cell line (HL60) with a GI_50_ value of 0.5 ± 0.02 µM [38]. In another study, Trykowska Konc et al. synthesized and evaluated 4-methyl coumarin derivatives as anti-cancer agents. Compound **VI** was found to be the most potent and inhibited the growth of leukemia CCRF-CEM (GI = 62.71 × 10^−5^ M), non-small-cell lung cancer HOP-92 (GI = 23.78 × 10^−5^ M), and colon cancer HCC-2998 (GI = 34.14 × 10^−5^ M) [39] (Figure 1).

*Cinnamomum* (cinnamon) is a genus belonging to the family Lauraceae. There are two main varieties of cinnamon: *Cinnamon zeylanicum* (Ceylon cinnamon) and *Cassia cinnamom* (Chinese cinnamon) [40,41]. Cinnamon was reported to possess numerous pharmacological activities such as antidiabetic, anti-Alzheimer, and antibacterial [40]. In addition, several biological investigations have proved the antiproliferative activity of the cinnamon extract against different malignant cells, including breast, lung, and ovarian carcinomas and leukemia [42,43]. These biological effects may have originated from cinnamon phytochemicals, mainly the volatile oil. The essential oil of *Cassia cinnamon* bark contains about 80–90% of cinnamaldehyde and a very little amount of eugenol, while that of *Cinnamomum zeylanicum* bark contains a lower amount of cinnamaldehyde (60–80%) and a considerable amount of eugenol [40]. It is noteworthy to mention that cinnamaldehyde exhibited an apoptotic effect on HepG2 with an IC_50_ value of 9.76 ± 0.67 μM and the HL60 cancer cell line [41,43].

Cinnamic acid is another component of cinnamon bark used as a fragrance and medicine. Cinnamic acid and its derivatives, either isolated from plant sources or synthesized, have received increasing attention due to its antioxidant, antiproliferative, and antiangiogenic activities [44]. The presence of a ubiquitous α- and β-unsaturated acid moiety characterized by its potential therapeutic effects as an anti-cancer agent enabled cinnamic acid derivatives acting on cancerous cells by various mechanisms of action. Therefore, these compounds are critical scaffolds in discovering novel anti-cancer agents [45].

The most efficient approaches to find lead compounds with remarkable biological activity is either the structural modification of biologically active natural products [38] or molecular hybridization to obtain a hybrid with better pharmacological activity. Based on these two approaches, several effective compounds have been developed to date wherein the final compounds had multiple pharmacophores with various binding affinities in different receptors [46,47].

**Figure 2:**
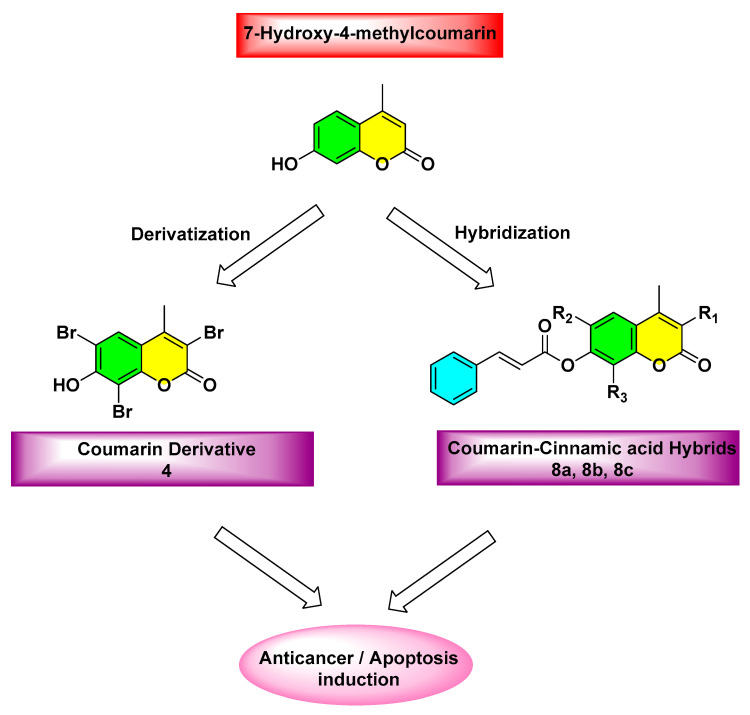
Design of coumarin derivatives and coumarin–cinnamic acid hybrids as anti-cancer agents.

Based on the aforementioned benefits, our aim was to synthesize coumarin derivatives and coumarin–cinnamic acid hybrids relying on the fundamental anti-cancer potentials of two bioactive scaffolds: 7-hydroxy-4-methylycoumarin and cinnamic acid (Figure 2), and to explore the cytotoxic activity of the synthesized derivatives on a panel of cancer cells. With an emphasis on HL60 and liver HepG2 cancer cell lines, the most active compounds were investigated for their inhibition of the PI3K/AKT pathway by gene and protein expression levels to prove and finally by the in-silico studies to confirm the mechanism of action.

## 2. Results and Discussion

### 2.1. Chemistry

**Scheme 1:**
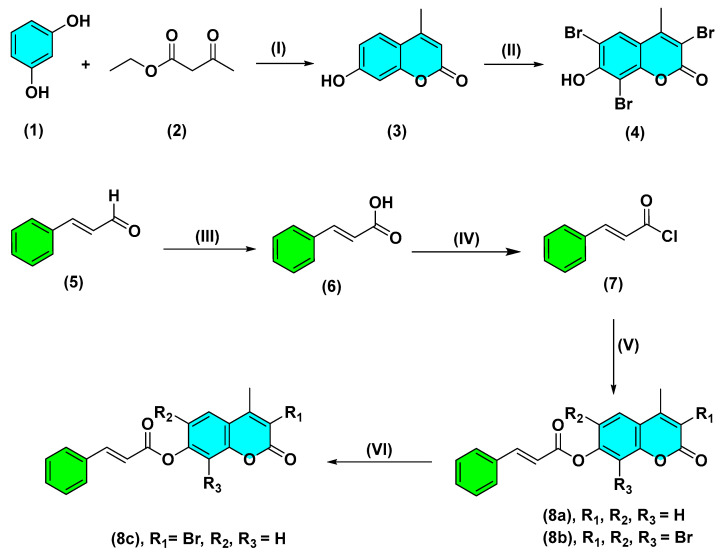
Reagents and conditions: (**I**) conc. H_2_SO_4_, reflux, (**II**) Br_2_/AcOH, 60 °C, (**III**) AgNO_3_, 30% H_2_O_2_, MeCN, 50 °C, (**IV**) SOCl_2_, reflux at 80 °C, CH_2_Cl_2_, 1.5 h, (**V**) compound (**3**) or (**4**), aq. NaOH/H_2_O_2_, DCM, RT, (**VI**) compound (**8a**), Br_2_/AcOH, 60 °C.

The synthetic steps of the target compounds are explained in Scheme 1. Full IR, ^1^H NMR, ^13^C NMR, and mass spectroscopic analyses were used to confirm the structures and purity of all synthesized compounds. First, the synthetic procedures started with the cyclo-condensation of resorcinol (**1**) and ethyl acetoacetate (**2**) in the presence of concentrated sulfuric acid as a catalyst to give 7-hydroxy-4-methylcoumarin (**3**) [48]. After that, the halogenation of compound (**3**) with bromine in glacial acetic acid led to a substitution reaction at positions 3, 6, and 8 to give 7-hydroxyl-3,6,8-tribromo-4-methylcoumarin (**4**), which was confirmed by comparing its NMR data with the literature values [49].

Cinnamic acid (**6**) is present in cinnamom bark (*Cassia cinnamon*) but in smaller amounts compared to cinnamaldehyde (**5**), which constitutes about 80–90% of *Cassia cinnamon* essential oil; hence, cinnamaldehyde could be easily isolated in considerably large amounts sufficient for chemical reactions. The essential oil of the bark was obtained by the hydro-distillation of cinnamon bark [50]. Cinnamadehyde was separated in the form of a sodium bisulfite additive product, then recovered by treatment with diluted HCl, followed by extraction with methylene chloride [51]. The aldehydic group was checked by testing with Schiff’s reagent. The structure of compound (**5**) was confirmed by co-chromatography with a standard sample of cinnamadehyde on precoated silica gel TLC plates developed by toluene: ethyl acetate (93:7 *v*/*v*) [52] and comparing its NMR values with the literature data [53]. Cinnamic acid (**6**) was obtained via the oxidation of its subsequent cinnamaldehyde (**5**) using the method adopted by Chakraborty and coworkers [54]. The structure of compound (**6**) was identified by comparing its NMR data with that reported for cinnamic acid [55]. Finally, (2*E*)-4-methyl-2-oxo-2H-chromen-7-yl cinnamate (**8a**) and (2*E*)-3,6,8-tribromo-4-methyl-2-oxo-2H-chromen-7-yl cinnamate (**8b**) were synthesized in good yields by two steps. The first step involved the reaction of cinnamic acid with thionyl chloride, followed by the reaction of the resulting cinnamoyl chloride (**7**), with compounds (**3**) and (**4**), respectively, in an aqueous solution of sodium hydroxide [56]. This was confirmed by the appearance of stretching bands of C-H olefinic of compounds (**8a**) and (**8b**) in the IR spectra and by the disappearance of phenolic hydroxyl stretching of compounds (**3**) and (**4**). The ^1^H NMR of compound (**8a**) exhibited two singlet signals; one of them was for the methyl group at *δ* (ppm) 2.35, and the other was for the proton at position 3 in the coumarin ring at *δ* (ppm) 6.11. In addition, the ^1^H NMR of compound (**8a**) showed signals of the protons of the phenyl moiety, coumarin ring and olefinic moiety as multiplets in the expected region of *δ* (ppm) 6.50–7.68.

The ^13^C NMR spectrum of compound (**8a**) showed the presence of two new carbon signals at *δ* (ppm) 168.24 and 161.43, due to the carbons of C=O and C-O groups, verifying the formation of compound (**8a**). The carbon signals of the coumarin, phenyl, and olefinic groups were observed through the expected chemical shift region at *δ* (ppm) 161.00–102.00. Carbon of the methyl group demonstrated a signal at *δ* (ppm) 18.52.

Concerning compound (**8b**), the ^1^H NMR spectrum showed the presence of two characteristic singlet signals at *δ* (ppm) 2.58 and 8.04 related to the protons of methyl group at position 4 and H-5 of the coumarin ring, respectively. The signals of aromatic and olefinic protons were observed through the expected chemical shifts in the region at *δ* (ppm) 6.49–7.67 and exhibited the expected integral values. The ^13^C NMR spectrum exhibited the occurrence of characteristic carbon signals at *δ* (ppm) 167.91 and 156.09 corresponding to C=O and C-O moieties, respectively. The carbon signals of coumarin and phenyl rings resonate at their usual positions (see experimental section). Moreover, the ^13^C NMR spectrum showed signals in the region at *δ* (ppm) 99.88 and 19.99 ppm, assigned to an olefinic carbon and a methyl group.

The halogenation of (2*E*)-4-methyl-2-oxo-2H-chromen-7-yl cinnamate (**8a**) with bromine in glacial acetic acid led to the formation of (2*E*)-3-bromo-4-methyl-2-oxo-2H-chromen-7-yl cinnamate (**8c**). The ^1^H NMR spectrum of compound (**8c**) showed the absence of the singlet signal at *δ* (ppm) 6.12 corresponding to H-3 of the coumarin ring, which appeared in compound (**8a**) clearly confirming the formation of compound (**8c**). In the ^1^H NMR spectrum of compound (**8c**), methyl protons resonated as singlet signals at *δ* (ppm) 2.55. The remaining proton signals were observed in the expected regions.

The ^13^C NMR spectrum of compound (**8c**) showed the presence of the characteristic carbon signal of the methyl group at *δ* (ppm) 19.77, which appeared in compound (**8a**) at *δ* (ppm) 18.52.

### 2.2. In Vitro Biological Evaluation

#### 2.2.1. Cytotoxic Screening against a Panel of Cancer Cell Lines

Cytotoxic activities of the synthesized derivatives were screened against leukemia (HL60), liver (HepG2), breast (MCF-7), and lung (A549) cancer cell lines and non-cancerous THLE2 and WISH cells at different concentrations [0.01–100 µM] for 48 h using the MTT assay (Table 1). Among the tested compounds, compound (**4**) exhibited cytotoxic effect against HL60, MCF-7, and A549 cancer cells, with IC_50_ values of 8.09, 3.26, and 9.34 µM, compared to staurosporine (IC_50_ = 7.48, 3.06, and 3.7 µM), respectively. While compound (**8b**), demonstrated cytotoxicity on HepG2, MCF-7, and A549 cancer cell lines with IC_50_ values of 13.14, 7.35, and 4.63µM, respectively, in comparison to staurosporine (IC_50_ = 10.24, 3.06, and 3.7 µM). Since compounds (**4**) and (**8b**) displayed the lowest IC_50_ values on the tested cancer cell lines and since our study was concerned with leukemia and liver cancer, these compounds (**4**) and (**8b**) were chosen for additional studies to investigate their mode of cytotoxicity on HL60 and HepG2 cancer cells, respectively.

#### 2.2.2. Investigation of Apoptotic Pathway

##### Flow Cytometric Analyses

FITC/Annexin-V-FITC/PI differential apoptosis/necrosis and DNA content-flow cytometry-aided cell-cycle analyses.

The apoptosis-inducing activity of compounds (**4**) (IC_50_ = 8.09 M, 48 h) and (**8b**) (IC_50_ = 13.14 M, 48 h) was investigated in HL60 and HepG2 cancer cells using cell-cycle analysis with cell populations at various stages of the cycle. After treatment with cytotoxic substances, the percentage of cells in each growth phase can be determined by investigating the cell cycle.

**Figure 3:**
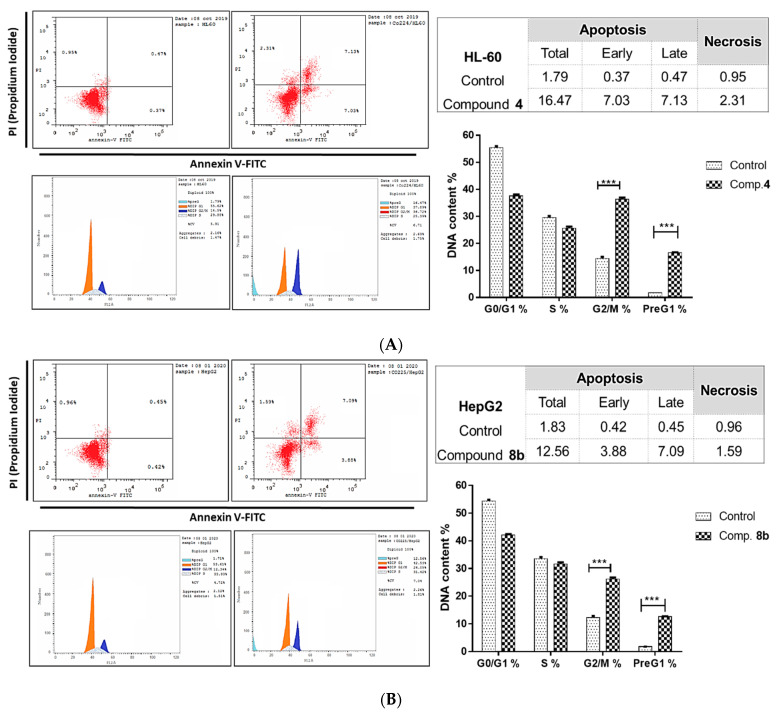
Cytogram showing annexin-V/propidium-iodide-stained “Quadrant charts show Q2-1 (necrotic cells, AV−/PI+), Q2-2 (late apoptotic cells, AV+/PI+), Q2-3 (normal cells, AV−/PI−), Q2-4 (early apoptotic cells, AV+/PI−)” and the cell-cycle distribution of both untreated and treated HL60 treated with compound **4** (IC_50_ = 8.09 μM, 48 h, **A**) and HepG2 cells treated with compound **8b** (IC_50_ = 13.14 μM, 48 h, **B**), with a bar chart representation of cell-population percentages in different cell-cycle phases. ***: *p* < 0.001.

Figure 3A shows that compound (**4**) significantly increased the death of leukemia cancer cells through apoptosis 9.2-fold (16.47% compared to 1.79% for the control). It also slightly induced necrosis-mediated cell death by 2.43-fold (2.31%, compared to 0.95% for the control). In addition, DNA flow cytometry was used to examine the cell-cycle kinetics of HL60 cancer cells treated with compound (**4**) to determine the compound’s phase interference with the cell cycle. It increased the G2/M cell (36.7%, compared to 14.5% for control) and pre-G1 (16.47%, compared to 1.79% for the control) population, and it decreased the cell population in the G0/G1 (37.89% compared to 55.62% for control).

Figure 3B shows that compound (**8b**) had a 6.88-fold effect on apoptotic liver cancer cell death (12.59%, compared to 1.83% for the control). Furthermore, it slightly induced necrosis-mediated cell death by 1.66-fold (1.59%, compared to 0.96% for the control). DNA flow cytometry was used to examine the phase-interface kinetics of the compound (**8b**) with the cell cycle after treatment with HepG2 cancer cells. It increased the G2/M cell (26.05%, compared to 12.34% for control) and pre-G1 (12.56%, compared to 1.71% for the control) population, and it decreased the cell population in the G0/G1 (42.53% compared to 53.6% for control).

Due to the cell-cycle arrest at G2/M, both compounds (**4**) and (**8b**) were able to inhibit the progression of HL60 and HepG2 cancer cells, which may be due to the induction of apoptosis by cell-cycle arrest at G2/M that degrades the genetic material.

##### Determination of Caspases 3/7 Activity

**Figure 4:**
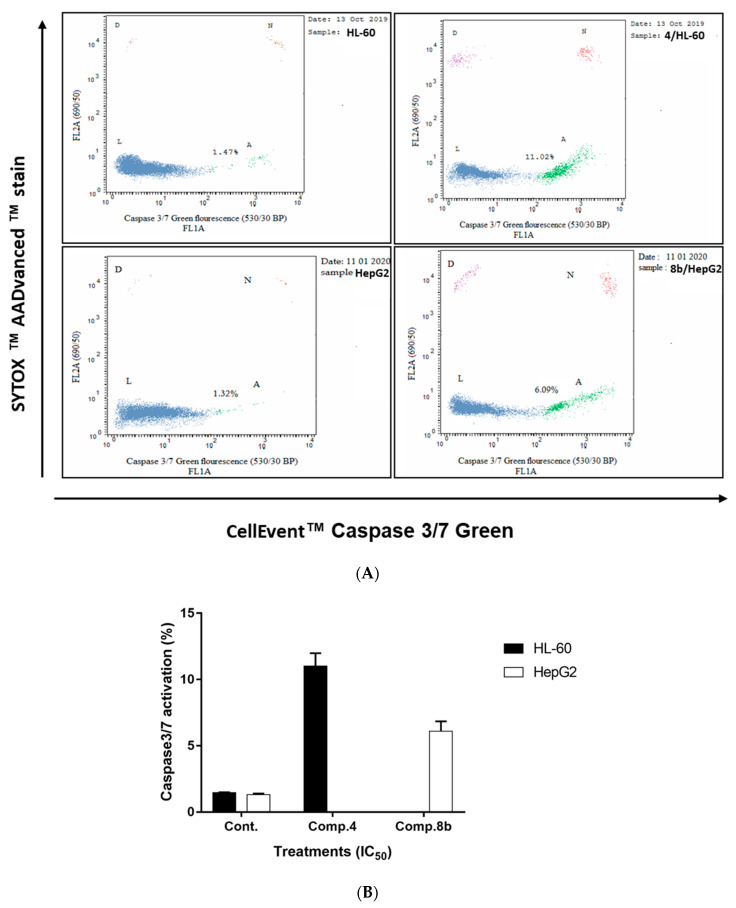
(**A**) Caspase inhibitory activity in cancer HL60 and HepG2 cells was treated with compounds (**4**) and (**8b**), respectively, for 48 h, using the “CellEvent^®^ Caspase 3/7 Green Flow Cytometry kit”, where “**L**, viable cells; **A**, apoptotic cells; **N**, necrotic cells; and **D**, dead cells”. (**B**) Graphical presentation of a comparison of apoptotic cancer cells due to active caspases 3/7 of the tested compounds. The data are expressed as the mean ± SEM of three independent experiments in triplicate.

Apoptosis is a form of cell death programmed by the family of cysteine protease. In response to the various cell death stimuli, a large irreversible proteolytic cascade is triggered and subsequently generated. The effector caspase activity has been evaluated to determine the apoptotic pathway initiated by the tested compounds using the “Cell Event™ Caspase 3/7 Green Detection kit”. As seen in Figure 4, compound (**4**) induced a higher percentage of HL60 cancer cell death via apoptosis, with a percentage of 11.02% than the untreated cells, 1.47%. Similarly, compound (**8b**) induced a higher percentage of apoptosis cells in HepG2 cells, with a percentage of 6.09%, than the untreated cells at 1.32%. Compounds (**4**) and (**8b**) showed that the apoptosis of cells was triggered by the activation of effectors 3 and 7 caspases.

##### Gene Expression Analysis for the Apoptosis-Related Genes

Both intrinsic and extrinsic genes (P53, Bax, Caspase 3, Caspase 7, PI3k, AKT, and Bcl-2) controlling apoptosis were screened for their relative mRNA expression using RT-PCR analysis to investigate the apoptosis-inducing pathway of the cytotoxic compound (**4**) (IC_50_ = 8.09 μM, 48 h) and compound (**8b**) (IC_50_ = 13.14 μM, 48 h), respectively, against HL60 and HepG2 cancer cells.

**Figure 5:**
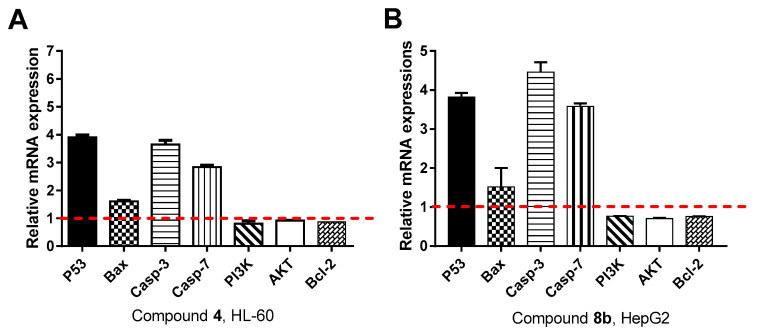
RT-PCR for the relative mRNA expression of treated HL60 treated with compound (**4**) (IC_50_ = 8.09 μM, 48 h, **A**) and HepG2 cells treated with compound (**8b**) (IC_50_ = 13.14 μM, 48 h, **B**) versus β-actin as a housekeeping gene (fold change of the untreated control = 1). The data are expressed as the mean ± SEM of three independent experiments.

The results in Figure 5A showed that compound (**4**)-treated HL60 cells induced the expression of pro-apoptotic genes: P53 as tumor suppressor gene by 3.8-fold, Bax by 1.63-fold, caspase 3 by 3.54-fold, caspase 7 by 2.88-fold relative to the mRNA expression of β-actin as housekeeping gene, while it inhibited the mRNA expression of the anti-apoptotic gene of Bcl-2 by 0.87-fold, PI3K by 0.88-fold, and AKT by 0.89-fold.

The results in Figure 5B demonstrated that compound (**8b**)-treated HepG2 cells strongly induced the expression of pro-apoptotic genes: P53 as tumor suppressor gene by 3.89-fold, Bax by 1.51-fold, caspase 3 by 4.63-fold, caspase 7 by 3.63-fold relative to the mRNA expression of β-actin as housekeeping gene, while it inhibited the mRNA expression of the anti-apoptotic gene of Bcl-2 by 0.75-fold, PI3K by 0.75-fold, and AKT by 0.72-fold.

Therefore, RT-PCR results confirmed the apoptosis-inducing activity of both compounds (**4**) and (**8b**) against HL60 and HepG2 cells, respectively, by their ability to increase the mRNA relative expression of pro-apoptotic genes and inhibit the mRNA for the anti-apoptotic genes.

##### Western Blotting and DNA Fragmentation

**Figure 6:**
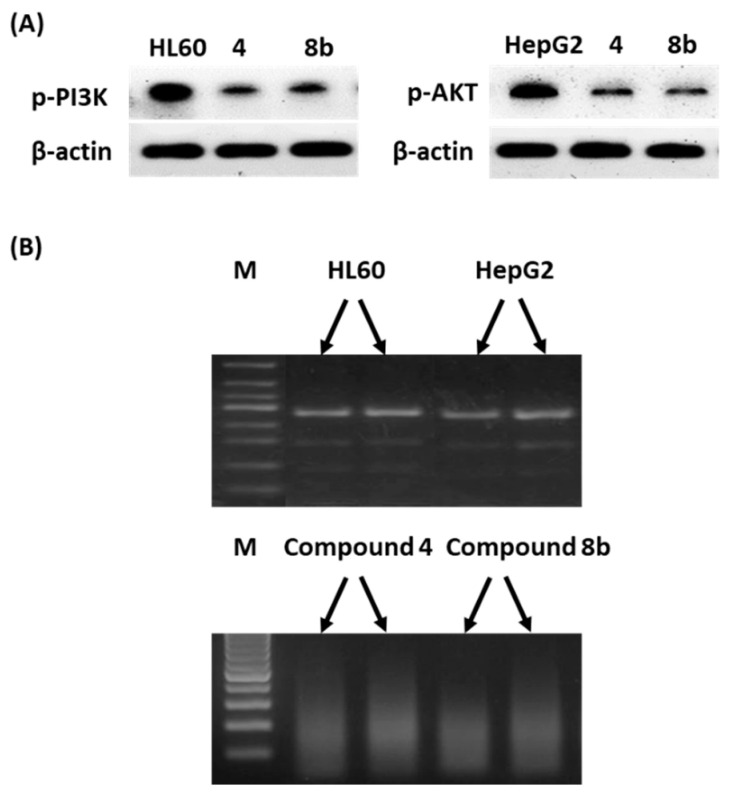
(**A**) Scanning densitometry Western blotting for p-PI3K and p-AKT proteins and (**B**) agarose gel electrophoresis DNA fragmentation for untreated and treated HL60 and HepG2 cells showing the loss of intact DNA fragments after exposure to compounds (**4**) and (**8b**) (M refers to the DNA ladder). Results are duplicate.

The apoptosis-inducing activity of compounds (**4**) and (**8b**) against HL60 and HepG2 cells through the inhibition of the PI3K/AKT signaling pathway was further confirmed by investigating Western blotting analysis and the DNA fragmentation of the expressed proteins. As shown in Figure 6A, following compound (**4**) and (**8b**) exposure, the levels of p-PI3K and p-Akt in HepG2 and HL60 cells were effectively suppressed with respect to control untreated cells. Loss of intact DNA fragments after exposure to compounds (**4**) and (**8b**) can be seen in agarose gel electrophoresis (Figure 6B). These results follow the relative gene expression of both PI3K/AKT genes and the apoptosis-inducing activity as a proposed mechanism.

Our results for the cytotoxic activity of both compounds (**4**) and (**8b**) against HL60 and HepG2 cancer cell lines, through apoptosis-inducing activity and the activation of the cell-cycle arrest at G2/M through flow cytometric analyses; gene and protein expression levels of PI3K/AKT proved the dual inhibitory action of the synthesized compounds. The inhibition of the PI3K/AKT pathways resulted in a caspase cascade, eventually leading to apoptosis [57,58,59].

### 2.3. In Silico Studies

#### 2.3.1. Computational Analysis

**Figure 7:**
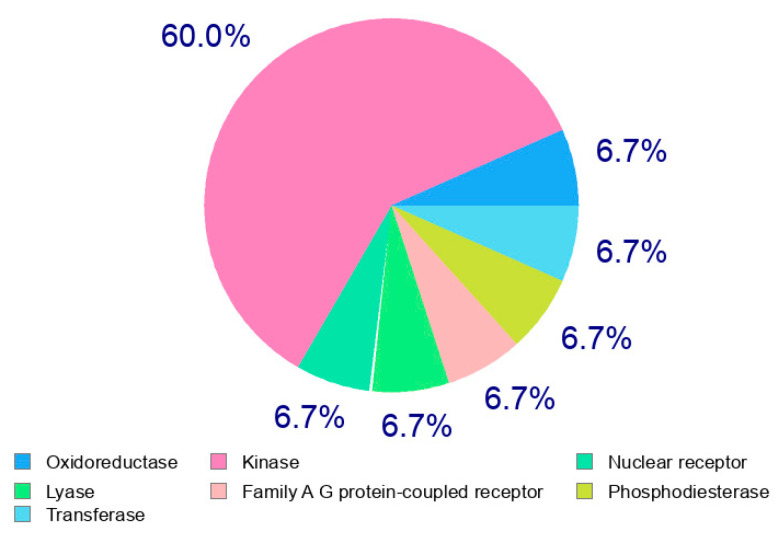
Target prediction of the proposed compounds using the SwissTargetPrediction Webtool.

The SwissTargetPrediction website tool was used to predict the most suitable target for the proposed compounds. It was found, as seen in Figure 7, that the anticipated derivatives may have a kinase receptor inhibitory action, with a probability of 60%. Other receptors were oxidoreductases, transferases, phosphodiesterases, family A G-protein coupled receptors, lyases, and nuclear receptors, each with a probability of 6.7%.

**Figure 8:**
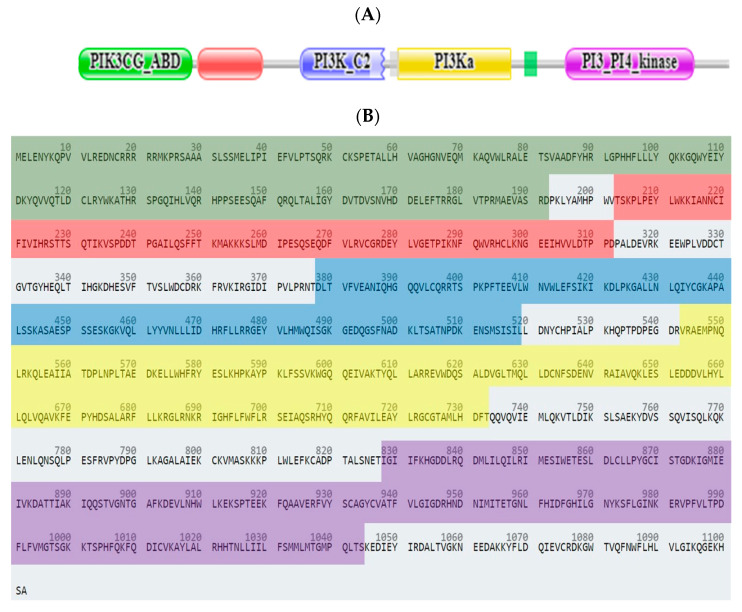
PI3K protein domains, (**A**) the arrangement of the Pfam domains, (**B**) the FASTA format of PI3K showing amino acid residues of 1. PIK3 catalytic subunit gamma adaptor-binding domain (green), 2. Ras-binding domain (red), 3. C2 domain (blue), 4. phosphoinositide 3-kinase family accessory domain (yellow), and 5. phosphoinositide 3/4-kinase domain (purple).

PI3K (phosphatidylinositol 4,5-bisphosphate 3-kinase catalytic subunit gamma isoform, Class I PI3K, Uniprot: P48736, Homo sapiens) is the protein that phosphorylates PtdIns(4,5)P2 (phosphatidylinositol 4,5-bisphosphate) to generate phosphatidylinositol 3,4,5-trisphosphate (PIP3). PIP3 plays a key role by engaging PH domain-containing proteins in the membrane, including AKT1, resulting in the activation of signaling cascades involved in cell growth and proliferation [60]. PI3K (Mass (Da)126,454) consists of 1102 amino acids that are arranged into five main domains (Figure 8A,B).

**Figure 9:**
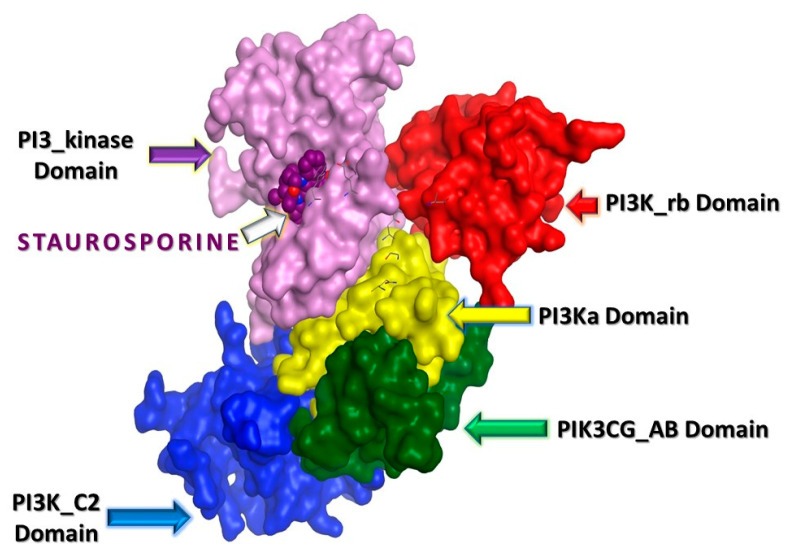
3D structure of the PI3K protein wherein there is a PIK3 catalytic subunit gamma adaptor-binding domain (green), 2. Ras-binding domain (red), 3. C2 domain (blue), 4. phosphoinositide 3-kinase family accessory domain (yellow), and 5. phosphoinositide 3/4-kinase domain (purple). Staurosporine is a PI3K inhibitor embedded in the phosphoinositide 3-kinase domain.

The PIK3 catalytic subunit gamma adaptor-binding domain (amino acid residues 1-192, PIK3CG_ABD) is the N-terminal domain that encompasses the adaptor-binding domain (ABD). The Ras-binding domain (amino acid residues 203-312, PI3K_rbd) is located in the N-termini. The C2 domain (amino acid residues 377-519, PI3K_C2) is about 116 amino acid residues and is located between the two copies of the C1 domain in protein kinase C and the protein kinase catalytic domain. The C2 domain is involved in calcium-dependent phospholipid binding. The Phosphoinositide 3-kinase family accessory domain (amino acid residues 543-733, PI3Ka domain) is a conserved domain in all PI3 and PI4-kinases. Phosphoinositide 3-kinase domain (amino acid residues 827-1044, PI3_PI4_kinase) can phosphorylate the 3â€™ position hydroxyl group of the inositol ring of phosphatidylinositol (PtdIns) [61] (Figure 9).

**Figure 10:**
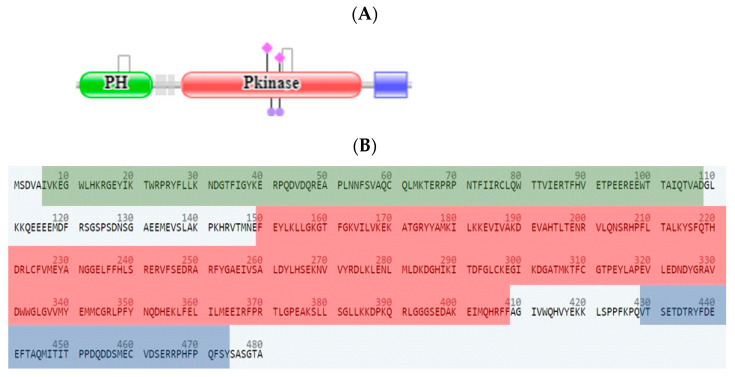
AKT1 protein domains, (**A**) the arrangement of the Pfam domains, (**B**) the FASTA format of AKT1 showing the amino acid residues of 1. Pleckstrin homology domain (green), 2. protein kinase domain (red), 3. protein kinase C terminal domain (blue).

**Figure 11:**
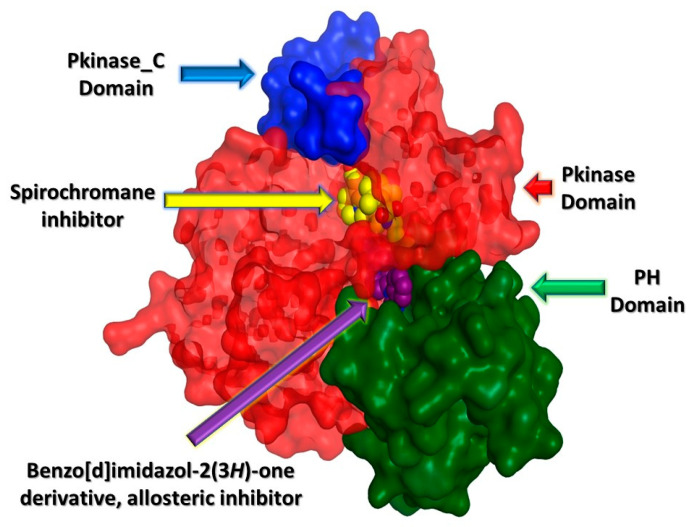
3D structure of the AKT1 protein wherein there is a Pleckstrin homology domain (green), protein kinase domain (red), and protein kinase C terminal domain (blue). AKT1 activity can be inhibited by two modes: inhibitors of the active site and allosteric inhibitors.

AKT1 (RAC-alpha serine/threonine-protein kinase, Uniprot: P31749, Homo sapiens) is a member of the serine/threonine-protein kinases (AKT1, AKT2, and AKT3), called AKT kinases, which are all closely related. The phosphorylation of MAP3K5 (apoptosis-signal-related kinase) by AKT regulates cell viability. Downregulation by RNA interference results in the induction of caspase-dependent apoptosis. AKT1 consists of 480 amino acids, arranged with three main domains [62] (Figure 10A,B); Pleckstrin homology domain (amino acid residues 6-108, PH domain), protein kinase domain (amino acid residues 150-408, PKinase domain), and protein kinase C terminal domain (amino acid residues 429-474, Pkinase_C domain) [63] (Figure 11).

The Pleckstrin homology domain is highly conserved in the different AKT isoforms (sequence homology between 76 and 84%) for its essential role in the interaction with membrane phospholipids, such as PIP3 produced by PI3K [63].

**Figure 12:**
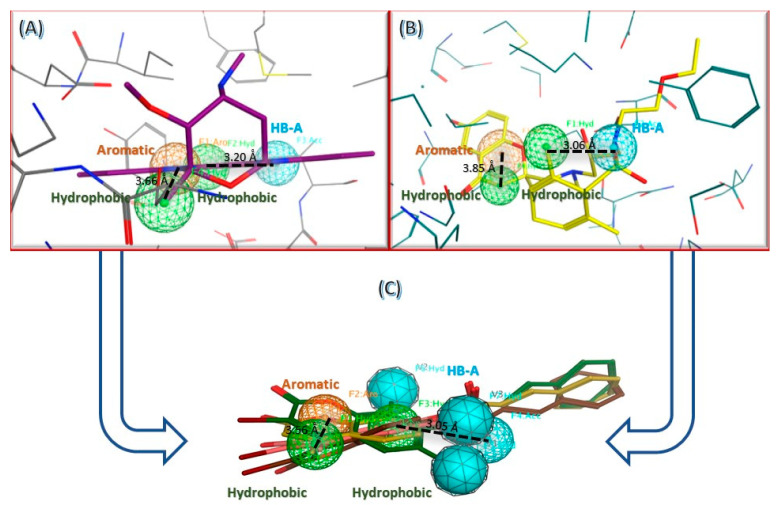
Common pharmacophoric features of the (**A**) staurosporine inhibitor (violet) of PI3K, (**B**) spirochromane inhibitor (yellow) of AKT1, and (**C**) superimposed structures of the proposed final compounds; hydrophobic features are represented as green contour spheres; aromatic features are represented as orange contour spheres, and HB_A features are represented as turquoise contour spheres. Additional HB_A features in the proposed compounds are represented as turquoise solid spheres.

From the literature survey, both coumarin and cinnamic acid scaffolds have potential PI3K/AKT1 signaling pathway inhibitory action [64,65,66,67], and either coumarin derivatization or combination with cinnamic acid proposed the final compounds that possess the main pharmacophoric features as the bound ligands in the active site of both PI3K and AKT1. Generally, PI3K and AKT1 inhibitors have a common “hydrophobic-aromatic ring-hydrophobic-HB_A” feature as previously reported [68,69]. Additionally, we found that the optimum length between the hydrophobic moiety and the aromatic ring should be in the range of 3.5–4.0 Å, while the optimum length between the other hydrophobic moiety and the HB_A group should be in the range of 3.0–3.5 Å. By superimposing the proposed compounds (**4**, **8a**, **8b**, **8c**), we found that all of the compounds fulfilled the required pharmacophoric features to act as PI3K/AKT1 inhibitors. Furthermore, compounds that had bromo-substitutions at positions 3,6,8 of the coumarin ring can form additional weak H-bonds or halogen bonds depending on the bond distances and angles with the adjacent amino acid residues as previously described [70,71]. This results in the stronger embedment of bromo-compounds in the kinase domains of the studied proteins (Figure 12).

To prove that the recommended candidates act as modulators or inhibitors of PI3K and AKT1, molecular docking was performed. From the PDB database, crystal structures with outliers and limited portions of the protein were excluded. The most suitable PDB codes were 1E8Z [72] and 3QKK [73] for PI3K and AKT1, respectively.

**Figure 13:**
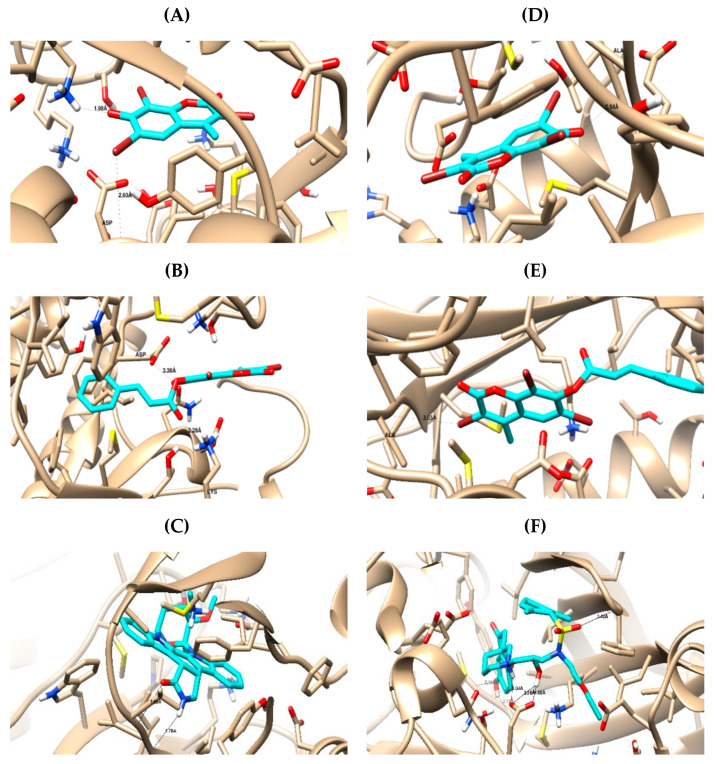
3D presentation of docked compounds (cyan-highlighted) inside the binding site of tested proteins; (**A**) compound **4** bound to PI3K key amino acid residues in the active site, (**B**) compound (**8b**) bound to PI3K key amino acid residues in the active site, (**C**) staurosporine (control) bound to PI3K key amino acid residues in the active site, (**D**) compound (**4**) bound to AKT key amino acid residues in the active site, (**E**) compound **8b** bound to AKT key amino acid residues in the active site, (**F**) spirochromane derivative (control) bound to AKT key amino acid residues in the active site.

Ligand–receptor interactions in the PI3K binding site for the synthesized compounds compared with the co-crystallized ligand staurosporine are summarized in Table S1. Compounds (**3**), (**4**), (**6**), (**8a**), (**8b**), and (**8c**) had H-bond interactions with Val882, which is the key amino acid in the PI3K receptor pocket [57]. Compounds (**4**) and (**8b**) were the best-docked compounds based on their low binding energy; H-bond interactions; and other hydrophobic interactions with Trp812, Met953, Met804, Phe961, Pro810, Ser806, Lys807, Ile963, Ile831, Ile879, Glu880, and Ala885 in the PI3 kinase domain (Figure 13a–c).

To prove the best binding mode for the synthesized compounds in AKT1, the compounds were docked in both the binding site and the allosteric site, and ligand–receptor interactions were compared with the co-crystallized ligands spirochromane derivative [73] and benzo[d]imidazole-2(3*H*)-one derivative [74], respectively. Binding interactions in the active site were stronger than in the allosteric site, where compounds (**3**), (**4**), (**6**), (**8a**), (**8b**), and (**8c**) had H-bond interactions with either Ala230 or Glu228, which are the key amino acids in the pocket of the AKT1 receptor (Table S1). Compounds (**4**) and (**8b**) were the best-docked compounds based on their low binding energy; H-bond interactions; and other hydrophobic interactions with Lys179, Thr291, Met227, Leu156, Phe438, Met281, Thr211, Glu234, Gly157, Met281 and Ala177 in the kinase domain (Figure 13d–f).

#### 2.3.2. In Silico ADME and Bioactivity Prediction

Drug-likeness is a complex balance of various molecular properties and structural features. These properties influence the behavior of a molecule in a living organism, including bioavailability, transport properties, affinity to proteins, reactivity, and many others. To identify the substructure features (which, in turn, determine physicochemical properties), the chemical structures of compounds (**4**) and (**8b**) were incorporated into the online SwissADME web tool. Physicochemical parameters such as number of heavy atoms; number of rotatable bonds; number of H-bond acceptors; number of H-bond donors; the fraction of carbon bond saturation (Csp3), i.e., the number of sp3 hybridized carbons/total carbon count; solubility (S) parameter LogS (Silicos-IT); lipophilicity parameter LogP predicted using the additive XLogP3 method; and molar refractivity for each compound were calculated versus both positive and negative controls (Table 2).

**Figure 14:**
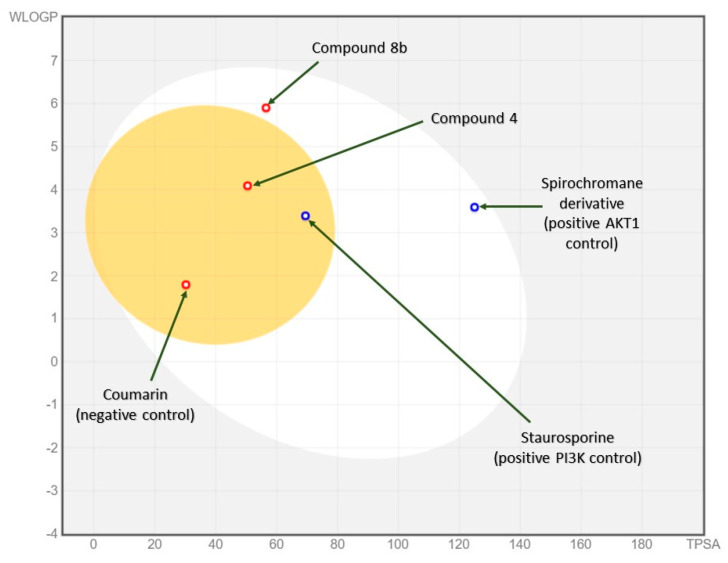
BOILED-Egg model: compound (**4**) and compound (**8b**).

The gastrointestinal absorption (high or low) and brain penetration (yes or no) of compound (**4**), compound (**8b**), and positive and negative controls were predicted and concluded via the BOILED-Egg model [75] (Figure 14). This model is based on two parameters: (1) the lipophilicity of the compounds under investigation, evaluated as a partition-coefficient (P) according to the Wildman–Crippen method [76] (WLogP), and (2) the compounds’ polarity, calculated as a topological polar surface area (tPSA) value. In this model, points located in the BOILED-Egg’s yolk are molecules predicted to passively permeate through the BBB, while points located in the BOILED-Egg’s white are molecules predicted to be passively absorbed by the GI tract.

The developed compounds were evaluated according to Veber’s rule-based method [77] to determine the drug-likeness for both of them, wherein a high probability of good bioavailability is more likely when there are 10 or fewer rotatable bonds and when the polar surface area is equal to or less than 140 Å^2^. From the data provided in Table 2, both compounds (**4**) and (**8b**) showed zero violations and, accordingly, can be considered good drug candidates for bioactivity studies.

**Figure 15:**
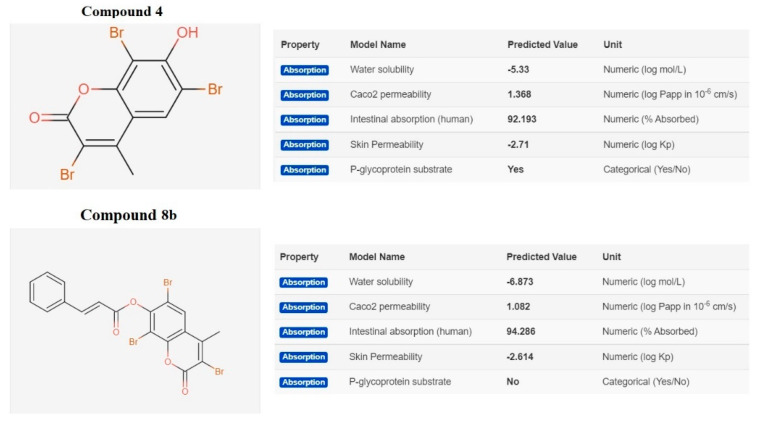
Compounds (**4**) and (**8b**) predicted P-glycoprotein substrates using the pkCSM website tool.

From pkCSM—pharmacokinetics [78], compound (**4**) and compound (**8b**) showed different predicted activities as P-glycoprotein substrates; compound (**4**) was predicted to act as a substrate for P-glycoprotein, while compound (**8b**) was not (Figure 15).

**Figure 16:**
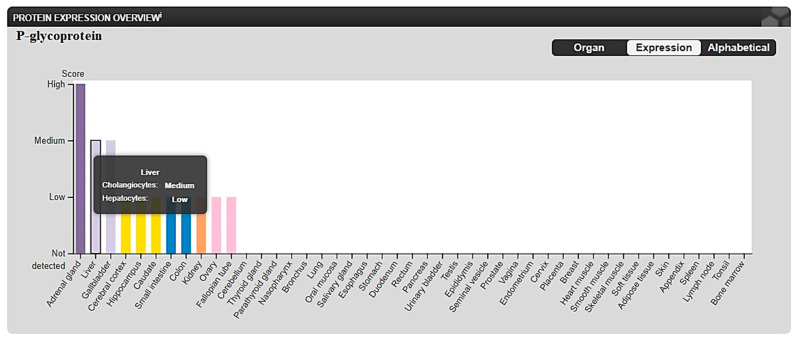
P-glycoprotein expression in different tissues.

The different selectivities of compounds (**4**) and (**8b**) in different cell lines, both HL60 or HepG2, may be explained due to the difference in predicted solubility and predicted P-glycoprotein binding, as P-glycoprotein expression differs between different cell types. P-glycoprotein shows medium-to-low expression in different liver (HepG2) cells but not expressed in leukocytes (HL60) (Figure 16) [79]. This may explain the lower activity of compound (**4**) (IC_50_ = 71.3 µM) than that of compound (**8b**) (IC_50_ = 13.14 µM) in HepG2 cell lines.

## 3. Materials and Methods

### 3.1. Chemistry

Reaction chemicals were purchased from Sigma Aldrich Chemical Co. (St. Louis, MO, USA) Pre-coated silica gel 60 F-254 plates (Merck, Darmstadt, Germany) were used for TLC. Spots were monitored by UV-light and *p*-anisaldehyde/sulfuric acid. Melting points were recorded on a Thomas Hoover Capillary melting point apparatus (Philadelphia, PA, USA) with a digital thermometer. IR spectra were obtained on a FT-IR Shimadzu 8300 spectrometer (Shimadzu, Tokyo, Japan). ^1^H NMR (400 MHz) and ^13^C NMR data were obtained on a Bruker NMR spectrometer in DMSO-d_6_, and chemical shifts were recorded in terms of parts per million (ppm) downfield from tetramethylsilane. Electron impact mass spectra (EI-MS) were recorded on a Shimadzu GCMS-QP 5050 A (Shimadzu, Tokyo, Japan) gas chromatograph–mass spectrometer (70 eV). Elemental analysis was done with an Elmentar, vario EL Germany Instrument at the Regional Centre for Mycology and Biotechnology, Faculty of Science, Al-Azhar University Perkin–Elmer 2400 (Waltham, MA, USA), and the results were within 0.4% of the calculated value.

#### 3.1.1. Synthesis of 7-Hydroxy-4-methylcoumarin (**3**)

Resorcinol (**1**) (10 mmol) and ethyl acetoacetate (**2**) (10 mmol) were refluxed in a water bath in the presence of concentrated sulfuric acid (2 mL) for 2 h. The reaction mixture was cooled and poured into water with continuous stirring. The resulting solid was filtered off, washed with water, dried (Na_2_SO_4_), and recrystallized from ethanol to give compound (**3**), a pale-yellow crystalline solid, with a yield of 87%. The NMR spectral data were in line with the literature [48] (Figures S1 and S2).

#### 3.1.2. Synthesis of 7-Hydroxy-3,6,8-tribromo-4-methylcoumarin (**4**)

To a solution of 7-hydroxy-4-methyl coumarin (**3**) (10 mmol) in 20 mL of glacial acetic acid was added 10 mL of bromine (30 mmol) in glacial acetic acid dropwise with stirring at 60 °C. After 5–10 min, the bromine color disappeared, and a yellow solution remained. At this point, 0.5–1 mL of the bromine–AcOH solution was added with continuous stirring at room temperature for 30–45 min. The reaction mixture was poured into water with continuous stirring, and the resulting product was filtered, washed with water, and dried (Na_2_SO_4_). Finally, the resulting product was purified by recrystallization from ethanol to give compound (**4**) as pale orange crystals, with a yield of 76%. The NMR spectral data were in line with the literature [49] (Figures S3 and S4).

#### 3.1.3. Isolation of cinnamaldehyde (**5**)

*Cassia cinnamon* bark was purchased from an Egyptian market and authenticated by the Faculty of Science, Suez Canal University, Ismailia, Egypt. A voucher specimen under registration no. (Cc-2019) was deposited in Pharmacognosy Department herbarium, Faculty of Pharmacy, Suez Canal University, Ismailia, Egypt.

Cinnamon oil was prepared by the hydro-distillation of *C. cinnamon* bark (500 g) in a Clevenger-type apparatus for 10 h. The distillate was transferred into a separating funnel. Then CH_2_Cl_2_ was added to extract the essential oil by solvent partitioning. The CH_2_Cl_2_ layer was transferred to a clean beaker. The extraction process was repeated, and all of the fractions were collected. The combined CH_2_Cl_2_ layers were washed with distilled water. The organic layer was dried (MgSO_4_). The CH_2_Cl_2_ extract was concentered under reduced pressure to yield 13.5 g of cinnamon oil.

To isolate cinnamaldehyde, a saturated aqueous solution of sodium bisulfite was added to cinnamon oil with vigorous shaking, and the resulted cinnamaldehyde-sodium bisulphite additive was filtered. The precipitate was washed with ethanol, then diethyl ether, followed by 5% HCl (250 mL) and refluxed at 60 °C for 30 min. After cooling, the reaction mixture was extracted with methylene chloride, then dried (MgSO_4_) to obtain cinnamaldehyde (**6**) as a yellow oily substance with a yield of 70%. Compound (**6**) was authenticated by co-chromatography with a standard cinnamaldehyde sample on silica gel TLC and visualized by UV and *p*-anisaldehyde/sulphuric acid spray reagent (R*_f_* 0.613, 93:7 *v*/*v* toluene-ethyl acetate, congruent to that of standard cinnamaldehyde). ^1^H NMR (CDCl_3_): *δ* 6.74 (dd, 1H, *J* = 15.6 and 7.7 Hz, H-2), 7.40–7.50 (m, 4H, 2× *meta*-Ar-H, *para*-Ar-H and H-3 olefinic), 7.53–7.59 (m, 2H, *ortho*-Ar-H), 9.70 (d, 1H, *J* = 7.7 Hz, H-1, aldehyde). ^13^C NMR (CDCl_3_): *δ* 128.57 (2C, *meta*-Ar-CH), 128.49 (C-2), 129.14 (2C, *ortho*-Ar-CH), 131.40 (*para*-Ar-CH), 134.04 (*ipso*-ArC), 152.9 (C-3), 194.07 (C-1) (Figures S5 and S6).

#### 3.1.4. Preparation of cinnamic acid (**6**)

To a stirred solution of 10 mol% of AgNO_3_ and cinnamaldehyde (**6**) (1 mmol) in acetonitrile (2 mL) was added 2 equivalents of 30% H_2_O_2_. The reaction mixture was heated slowly at 50 °C. The reaction was quenched with a cold aqueous solution of 10% Na_2_S_2_O_3_ and then extracted with CH_2_Cl_2_. The organic layer was separated and evaporated under reduced pressure. The residue obtained was alkalinized with an aqueous solution of NaHCO_3_ and extracted with CH_2_Cl_2_. The aqueous layer was acidified by 2N HCl, then extracted with CH_2_Cl_2_. The organic layer was concentrated under vacuum. The crude product was purified by silica gel column chromatography to obtain colorless crystals of cinnamic acid (**7**), with a yield of 85%, m.p. 133 °C, ^1^H NMR (DMSO d_6_): *δ* 6.55 (d, 1H, *J* = 15.6, H-2), 7.42–7.43 (m, 3H, 2×meta-Ar-H and para-Ar-H), 7.61 (d, 1H, *J* = 15.6, H-3) 7.69–7.70 (m, 2H, *ortho*-Ar-H), and 12.44 (s, 1H). ^13^C NMR (DMSO d_6_): *δ* 119.31 (C-2), 128.68 (2C, *meta*-Ar-CH), 129.56 (2C, *ortho*-Ar-CH), 131.00 (*para*-Ar-CH), 134.40 (*ipso*-ArC), 144.01 (C-3), 168.08 (C-1) (Figures S7 and S8).

#### 3.1.5. General procedure for the synthesis of compounds (**8a**, **8b**)

A mixture of cinnamic acid (**6**) (0.83 mmol) and thionyl chloride (3.5 mmol) was refluxed for 4 h (monitored by TLC). The product (cinnamoyl chloride (**7**)) was used immediately for the next step without any further purification.

To a chilled solution of cinnamoyl chloride (**7**) (1 mmol) in dichloromethane (1 mL) was added dropwise over a period of 30 min with stirring an aq. solution of sodium hydroxide (2 mmol) and coumarin (2 mmol). The mixture was stirred for 1.5 h at room temperature. The product was obtained by filtration, washed with cooled water, and recrystallized from aqueous ethanol.

##### (2E)-4-Methyl-2-oxo-2H-chromen-7-yl Cinnamate (**8a**)

Compound (**8a**) was obtained as yellow crystals, yield 81%, m.p. 96 °C, IR (KBr) ν_max_ 1725–1705 (C=O), 1627, 1605, 1595 (C=C), 1377 (CH_3_) 1175, 1068 and 1025 (C-O) cm^−1^. ^1^H NMR (DMSO d_6_): *δ* 2.35 (s, 3H, CH_3_), 6.11 (s, 1H, C3-H), 6.53 (d, 1H, *J* = 16 Hz, C=CH-CO), 6.72 (d, 1H, *J* = 4 Hz, C8-H), 6.81 (dd, 1H, *J* = 4, 8 Hz, C6-H), 7.40–7.42 (m, 3H, Ar-H), 7.56 (d, 1H, *J* = 8 Hz, C5-H), 7.59 (d, 1H, *J* = 8 Hz, Ph-CH=C) 7.65–7.68 (m, 2H, Ar-H). ^13^C NMR (DMSO-d_6_): *δ* 168.24, 161.43, 155.18 (2), 154.18, 144.33, 134.58, 130.69, 129.38 (2), 128.56 (2), 127.31, 119.69 (2), 113.32, 112.14, 110.62, 18.52. MS (m/z) = 306 (M^+^). Anal. Calcd. for C_19_H_14_O_4_ (306): C, 74.50; H, 4.61. Found: C, 74.66; H, 4.39 (Figures S9–S14).

##### (2E)-3,6,8-Tribromo-4-methyl-2-oxo-2H-chromen-7-yl Cinnamate (**8b**)

Compound (**8b**) was obtained as pale-yellow crystals, yield 82%, m.p. 210 °C, IR (KBr) ν_max_ 1731–1700 (C=O), 1627, 1589 (C=C), 1377 (CH_3_), 1088 and 1026 (C-O) cm^−1^. ^1^H NMR (DMSO-d_6_): *δ* 2.58 (s, 3H, CH_3_), 6.51 (d, 1H, *J* = 16 Hz, C=CH-CO (olefinic-H), 7.41–7.42 (m, 3H, Ar-H), 7.59 (d, 1H, *J* = 16 Hz, Ph-CH=C), 7.66–7.67 (m, 2H, Ar-H), 8.04 (s, 1H, C5-H). ^13^C NMR (DMSO-d_6_): *δ* 167.91, 156.09, 154.80, 151.37, 149.50, 144.30, 134.73, 130.61, 129.33 (2), 128.69, 128.59 (2), 119.74, 114.74, 110.12, 108.28, 99. 88, 19.99. MS (m/z) = 540 (M^+^). Anal. Calcd. for C_19_H_11_Br_3_O_4_ (540): C, 42.03; H, 2.04. Found: C, 42.33; H, 1.92 (Figures S15 and S16).

#### 3.1.6. Synthesis of (2E)-3-bromo-4-methyl-2-oxo-2H-chromen-7-yl cinnamate (**8c**)

Ten mL of bromine (10 mmol) in glacial acetic acid was added dropwise with continuous stirring to a solution of 3-(4-methyl coumarin-7-yloxy)-3-phenyl acrylic acid (**8a**) (10 mmol) in 15 mL of glacial acetic acid at 60 °C. After 5–10 min, the bromine color disappeared, and a yellow solution remained. At this point, 0.5–1 mL of a bromine–AcOH solution was added with stirring at room temperature for 30–45 min. The reaction mixture was poured into water with stirring, and the solid formed was filtered, washed with water, and dried (Na_2_SO_4_). Finally, the product was recrystallized from methanol to give compound **(8c)** as pale-yellow crystals, with a yield of 61%, m.p. 170 °C. IR (KBr) ν_max_ 1725–1700 (br. C=O), 1617, 1583(C=C), 1378 (CH_3_), 1095 and 1078 (C-O) cm^−1^. ^1^H NMR (DMSO-d_6_): *δ* 2.55 (s, 3H, CH_3_), 6.53 (d, 1H, *J* = 16 Hz, C=CH-CO), 6.70 (d, 1H, *J* = 4 Hz, C8-H), 6.79 (dd, 1H, *J* = 4, 8 Hz, C6-H), 7.37–7.40 (m, 3H, Ar-H), 7.54 (d, 1H, *J* = 8 Hz, C5-H), 7.57 (d, 1H, *J* = 8 Hz, Ph-CH=C) 7.63–7.66 (m, 2H, Ar-H). ^13^C NMR (DMSO-d_6_): *δ* 161.88, 158.78, 157.90, 153.58, 152.49, 152.30, 150.43, 130.69, 129.36, 128.66, 127.79, 126.27, 114.35, 112.22, 109.08, 108.63, 102.52, 97.24, 19.77. MS (m/z) = 384 (M^+^, unstable). Anal. Calcd. for C_19_H_13_BrO_4_ (384) C, 59.24; H, 3.40. Found: C, 59.11; H, 3.08 (Figures S17 and S18).

### 3.2. In Vitro Biological Assays

#### 3.2.1. Cell culture

Human promyelocytic leukemia ”HL-60”, human hepatocellular carcinoma “HepG2”, human breast cancer “MCF-7”, human lung cancer “A549” cell lines, and two normal cell lines, THLE-2 as normal adult liver epithelial cells and WISH as normal epithelial cells, were obtained from American Type Culture Collection “Rockville, MD, USA”. All cell lines were cultured in an RPMI medium from “Invitrogen/Life Technologies” at 37 °C in a humid atmosphere with 5% CO_2_ according to standard tissue culture protocols [80].

#### 3.2.2. Cytotoxic Activity Using MTT Assay

The cytotoxic activity of the tested compounds (**3**, **4**, **6**, **8a**, **8b,** and **8c**) was determined by the MTT assay [81,82]. According to the manufacturer’s protocol, the cells were seeded in 96-well plates at a concentration of 5 × 10^4^ cells per well (200 µL). Different concentrations of the tested compounds were used to test the cytotoxicity. There were three replicates of each concentration, and the standard control was staurosporine, with vehicle DMSO used as the blank. After 48 h of incubation at 37 °C, 10 L of the MTT stock solution was added to each well and incubated again for 4 h. Finally, the optical absorbances at 570 nm were measured, and cell survival was calculated using the following equation: “percentage of cell viability = $\;\frac{Asapmple}{Acontrol}\times 100”$. A non-linear regression of curve fit was used to calculate the IC_50_.

#### 3.2.3. Investigation of the Apoptotic Pathway

##### Flow Cytometric Analysis

HL60 and HepG2 cancer cells were treated with compound (**4**) (IC_50_ = 8.09 μM, 48 h) and compound (**8b**) (IC_50_ = 13.14 μM, 48 h), respectively, compared to control, then the apoptotic mechanistic mode of action was investigated through flow cytometric analyses (ACEA Biosciences Inc., San Diego, CA, USA), as previously described [83]. (See supplementary information for the detailed methodology of flow cytometric analyses, including “FITC/Annexin-V-FITC/PI differential apoptosis/necrosis”, “DNA content-flow cytometry aided cell cycle”, and “Caspase 3/7 green flow cytometry”).

##### Gene Expression Using RT-PCR Analysis

After 48 h of treatment with compound (**4**) and compound (**8b**) (IC_50_ = 8.09 M and 13.14 µM, respectively), total RNA was extracted from both treated and untreated cancer cells using Qiagen RNA extraction (GmbH, Hilden, Germany). The synthesis of cDNA was then performed, followed by the qPCR test in one tube. The primer sequence pairs (Table 3) were selected for the tested genes “P53, BAX, CASP-3, -9, PI3K, AKT, BCL2” and β-actin as housekeeping gene. Cycle thresholds (Ct) and Ct were used to calculate the relative quantities of each gene tested, as previously described [83].

##### Western Blottting

Western blotting analysis was conducted using untreated and treated HepG2 and HL60 cells. Cells were washed in PBS and lysed in a boiling sample buffer (62.5 mM Tris-HCl pH 6.8, 1% SDS, 10% glycerol, and 5% β-mercaptoethanol) for sodium dodecyl sulfate polyacrylamide gel electrophoresis (SDS-PAGE). The lysates were boiled for 5 min with a lamellae buffer, and the proteins were separated by SDS-PAGE and transferred to an Immobilon membrane (Millipore, Burlington, MA, USA). After incubation in 5% non-fat dry milk, Tris-HCL, 0.1% Tween 20 for 1 h, p-PI3K, and p-AKT, primary antibodies were added to one of the membranes containing specimen samples and incubated at 4 °C overnight. Appropriate secondary antibodies were incubated for 2 hr at room temperature. After being washed twice n 1× TBS-T, densitometric analysis of the immunoblots was performed to quantify the amounts of p-PI3K and p-AKT against the control sample by total protein normalization using Image analysis software on the ChemiDoc MP imaging system (version 3) produced by Bio-Rad (Hercules, CA, USA) [64].

### 3.3. In Silico Studies

Target prediction was done using the SwissTargetPrediction web tool [83].

#### 3.3.1. Molecular Docking

Two PDB codes for the molecular targets in the proposed cytotoxic mechanism were selected. Both 1E8Z and 3QKK were chosen as crystal structures for PI3K and AKT receptors, respectively. The docking program involved in our evaluation was AutoDock Vina [84,85], following a multiple ligands docking protocol [86].

The grid box size was prepared based on the active residues of the kinase domains’ ATP binding sites. After docking simulation, poses with the highest negative binding free energy (kcal/mol) were selected as the best pose for the corresponding ligand binding. The visualization of the 3D receptor binding site, the disposition of the original (co-crystallized) ligand, and the main ligand receptor interaction in terms of hydrogen bonding and lipophilic interaction with the key amino acid residues were done using Chimera software [87].

#### 3.3.2. In Silico Physicochemical Descriptors, Pharmacokinetic Properties, and Bioactivity Prediction

The estimation of physicochemical properties, pharmacokinetics, and drug-likeness in silico was performed using SwissADME and PKCSM web tools [78,88].

## 4. Conclusions

In conclusion, we have synthesized new derivatives of (2E)-4-methyl-2-oxo-2H-chromen-7-yl cinnamate (**8a**), (**8b**), and (**8c**) in addition to the known compound: 7-hydroxy-3,6,8-tribromo-4-methylcoumarin (**4**). The chemical structures of the synthesized compounds were proven by IR, NMR, and MS spectral analyses. Using the MTT assay, the cytotoxicity of all of the synthesized compounds, as well as their precursors, was evaluated on a panel of cancerous cells, among which compounds (**4**) and (**8b**) were the most active ones on the tested cancer cell lines. Compound (**4**) had a cytotoxic effect against HL60 cells, with an IC_50_ value of 8.09 µM, while compound (**8b**) had an activity against HepG2 cells, with IC_50_ value of 13.14 µM. Since our resaerch was directed towards leukemia and liver cancer due their high resistance to chemotherapy and increased incidence rate, the proposed mechanism of the cytotoxicity of compounds (**4**) and (**8b**) on HL60 and HepG2, respectively, was inspected using cell-cycle analysis, real-time PCR, DNA fragmentation, and Western blotting analyses. Our results revealed that both compounds (**4**) and (**8b**) displayed cytotoxic activity through the PI3K/AKT signaling pathway, which was further confirmed by molecular docking studies.

## Figures and Tables

**Table 1:** Summary of the IC_50_ (µM) values of the tested derivatives against a panel of cancer cells.

Compound No.	IC_50_ (µM) ± SEM *^#^					
	Leukemia Cancer HL60	Liver Cancer HepG2	Breast Cancer MCF-7	Lung Cancer A549	Normal Liver THLE2	Normal WISH Cells
**3**	42.55 ± 0.22	95.65 ± 1.13	29.3 ± 1.02	16.5 ± 1.98	-	-
**4**	8.09 ± 0.13	71.3 ± 1.52	3.26 ± 0.12	9.34 ± 0.34	116.6 ± 3.34	89.6 ± 2.34
**6**	88.21 ± 0.51	36.58 ± 0.19	28.6 ± 0.91	27.6 ± 1.95	-	-
**8a**	34.8 ± 0.64	75.2 ± 1.39	39.3 ± 1.69	19.6 ± 0.89	-	-
**8b**	68.95 ± 2.14	13.14 ± 0.22	7.35 ± 0.67	4.63 ± 0.16	86.5 ± 2.16	96.8 ± 3.21
**8c**	48.1 ± 0.88	17.4 ± 0.19	67.3 ± 2.03	24.3 ± 0.37	124 ± 2.12	-
**Staurosporine**	7.48 ± 0.11	10.24 ± 0.16	3.06 ± 0.42	3.7 ± 0.09	73.2 ± 2.8	69.7 ± 2.4

* Values are mean ± SEM of three independent replicates. ^#^ “IC_50_ values are calculated through EXCEL using a non-linear regression curve fit of sigmoidal dose–response inhibition”.

**Table 2:** Predicted physicochemical descriptors of the synthesized compound (**4**) and compound (**8b**).

Compound	(4)	(8b)	Staurosporine (Positive PI3K Control)	Spirochromane Derivative (Positive AKT1 Control)	Coumarin (Negative Control)
Heavy atoms	16	26	35	38	11
Rotatable bonds	0	4	2	10	0
H-bond donors	1	0	2	2	0
H-bond acceptors	3	4	4	9	2
Fraction Csp3	0.10	0.05	0.32	0.61	0.00
Silicos-IT	−5.89	−8.62	−7.59	−5.08	−3.59
XLogP3	3.75	5.94	3.24	1.66	1.39
Molar refractivity	72.57	111.66	139.39	150.15	42.48

**Table 3:** Forward and reverse primers used in gene expression analysis.

Gene	Forward	Reverse
**P53**	5′-CCCCTCCTGGCCCCTGTCATCTTC-3′	5′-GCAGCGCCTCACAACCTCCGTCAT-3′
**BAX**	5′-GTTTCATCCAGGATCGAGCAG-3′	5′-CATCTTCTTCCAGATGGTGA-3′
**CASP3**	5′-TGGCCCTGAAATACGAAGTC-3′	5′-GGCAGTAGTCGACTCTGAAG-3′
**CASP9**	5′-CGAACTAACAGGCAAGCAGC-3′	5′-ACCTCACCAAATCCTCCAGAAC-3′
**PI3K**	5′-CTCTCCTGTGCTGGCTACTGT-3′	5′-GCTCTCGGTTGATTCCAAACT-3′
**AKT**	5′-GGACAAGGACGGGCACATTA-3′	5′-CGACCGCACATCATCTCGTA-3′
**BCL2**	5′-CCTGTGGATGACTGAGTACC-3′	5′-GAGACAGCCAGGAGAAATCA-3′
**β-actin**	5′-GTGACATCCACACCCAGAGG-3′	5′-ACAGGATGTCAAAACTGCCC-3′

## Data Availability

Data is contained within the article and Supplementary Materials.

## Supplementary Materials

The following are available online at https://www.mdpi.com/article/10.3390/molecules27196709/s1, Table S1: Docked interaction analysis of synthesized ligands screened with the two proteins (PI3K and AKT1); Figure S1: ^1^H NMR of compound (**3**); Figure S2: ^13^C NMR of compound (**3**); Figure S3: ^1^H NMR of compound (**4**); Figure S4: ^13^C NMR of compound (**4**); Figure S5: ^1^H NMR of Cinnamaldehyde (**5**); Figure S6: ^13^C NMR of Cinnamaldehyde (**5**); Figure S7: ^1^H NMR of Cinnamic Acid (**6**); Figure S8: ^13^C NMR of Cinnamic Acid (**6**); Figure S9: ^1^H NMR of compound (**8a**); Figure S10: ^13^C NMR of compound (**8a**); Figure S11: NOSY of compound (**8a**); Figure S12: COSY of compound (**8a**); Figure S13: HMBC of compound (**8a**); Figure S14: HSQC of compound (**8a**); Figure S15: ^1^H NMR of compound (8b); Figure S16: ^13^C NMR of compound (**8b**); Figure S17: ^1^H NMR of compound (**8c**); Figure S18: ^13^C NMR of compound (**8c**).
