# Supplementary material for: Design and Synthesis of Coumarin Derivatives as Cytotoxic Agents through PI3K/AKT Signaling Pathway Inhibition in HL60 and HepG2 Cancer Cells
Source: Molecules. 2022 Oct 9;27(19):6709. doi: 10.3390/molecules27196709 (PMC9571264; doi:10.3390/molecules27196709)
Supplement: Supplementary file 1 [file molecules-27-06709-s001.zip › molecules-1862399-supplementary.pdf]

Design and Synthesis of Coumarin Derivatives as Cytotoxic Agents through PI3K/AKT Signaling Pathway Inhibition in HL60 and HepG2 Cancer Cells

Table of content

Item	Page No.
Fig. S1. ^{1}H NMR of compound (3)	2
Fig. S2. ^{13}C NMR of compound (3)	2
Fig. S3. ^{1}H NMR of compound (4)	3
Fig. S4. ^{13}C NMR of compound (4)	3
Fig. S5. ^{1}H NMR of Cinnamaldehyde (5)	4
Fig. S6. ^{13}C NMR of Cinnamaldehyde (5)	4
Fig. S7. ^{1}H NMR of Cinnamic Acid (6)	5
Fig. S8. ^{13}C NMR of Cinnamic Acid (6)	5
Fig. S9. ^{1}H NMR of compound (8a)	6
Fig. S10. ^{13}C NMR of compound (8a)	6
Fig. S11. NOSY of compound (8a)	7
Fig. S12. COSY of compound (8a)	7
Fig. S13. HMBC of compound (8a)	8
Fig. S14. HSQC of compound (8a)	8
Fig. S15. ^{1}H NMR of compound (8b)	9
Fig. S16. ^{13}C NMR of compound (8b)	9
Fig. S17. ^{1}H NMR of compound (8c)	10
Fig. S18. ^{13}C NMR of compound (8c)	10
Supplementary materials for detailed methodology	11
2.2.2.1. Flow cytometric analysis	11
2.2.2.3. Immunoblotting (Western blotting) for PI3K and AKT	12
2.3.1. Table S1. Docked interaction analysis of synthesized ligands screened with the two proteins (PI3K and AKT1).	12

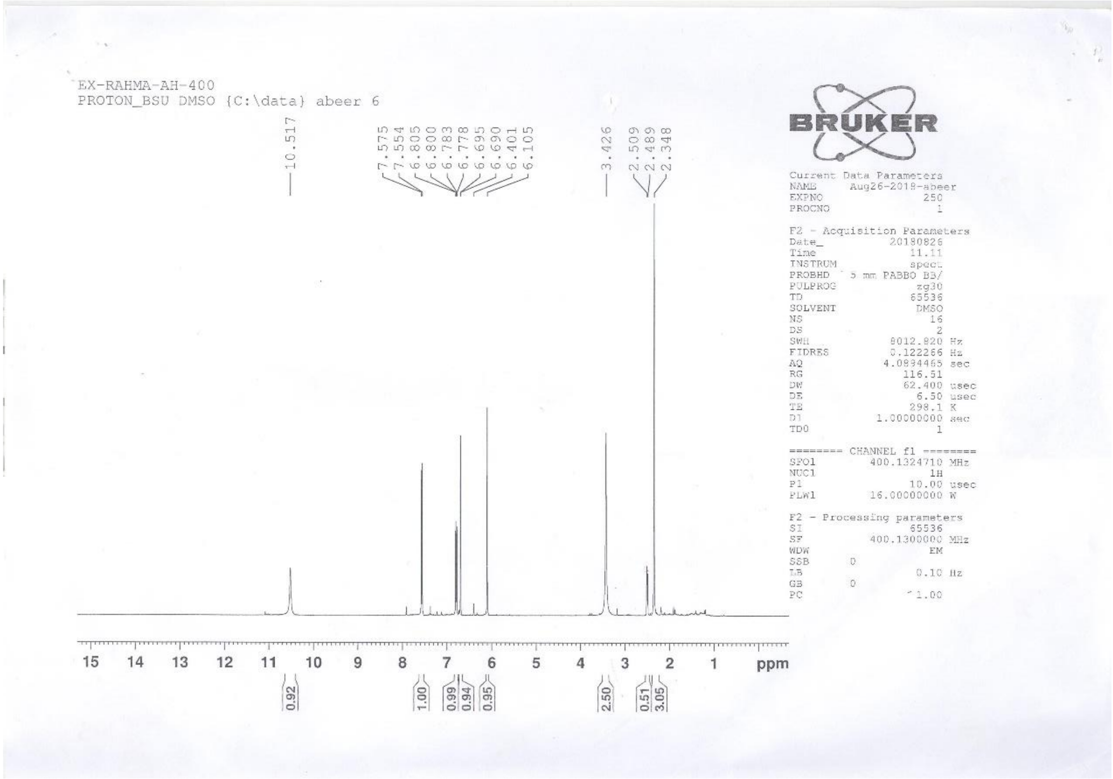

Fig. S1. ¹H NMR of Compound (3)

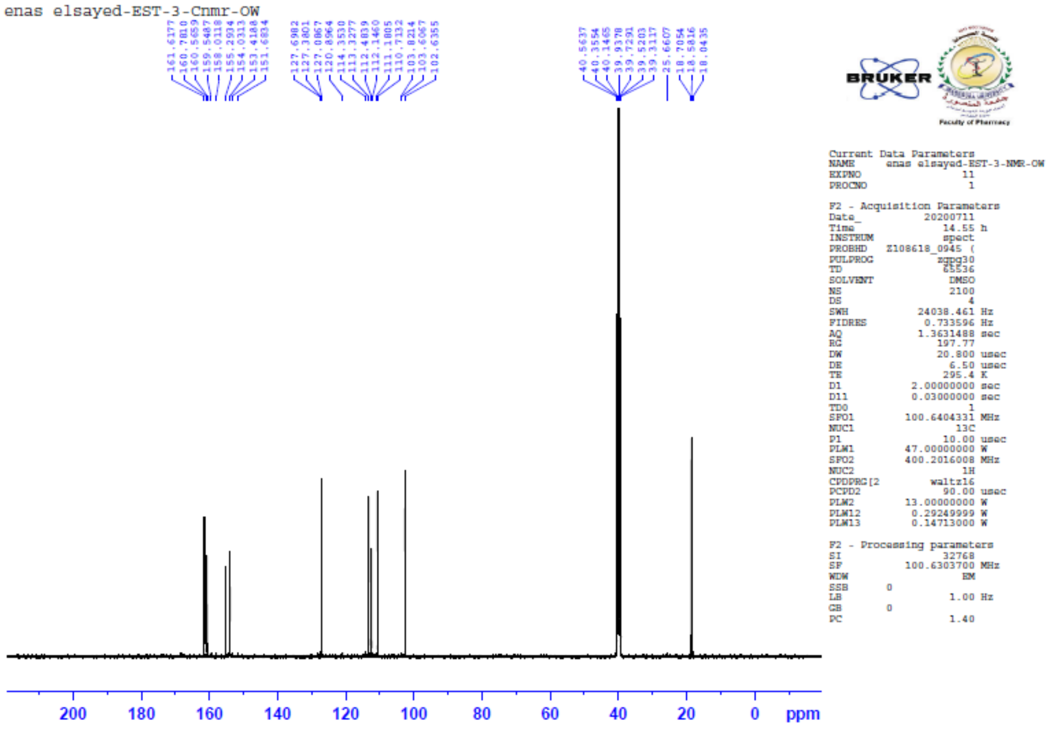

Fig. S2. ¹³C NMR of Compound (3)

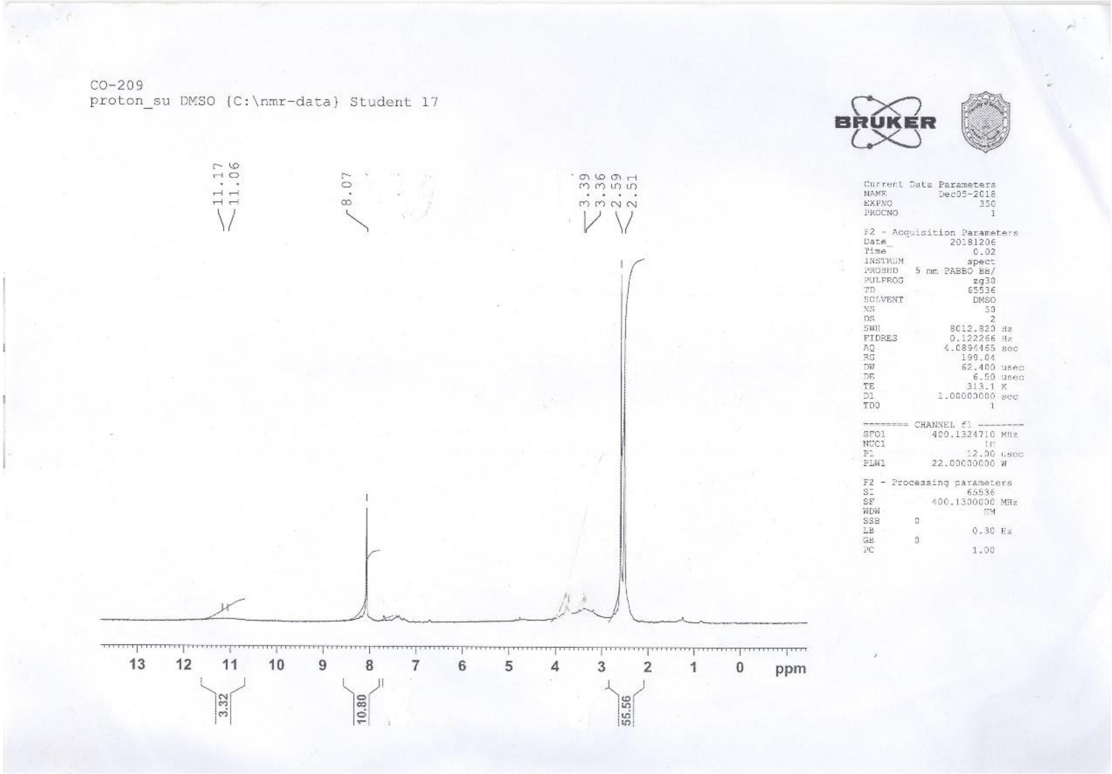

Fig. S3. ^{1}H NMR of Compound (4)

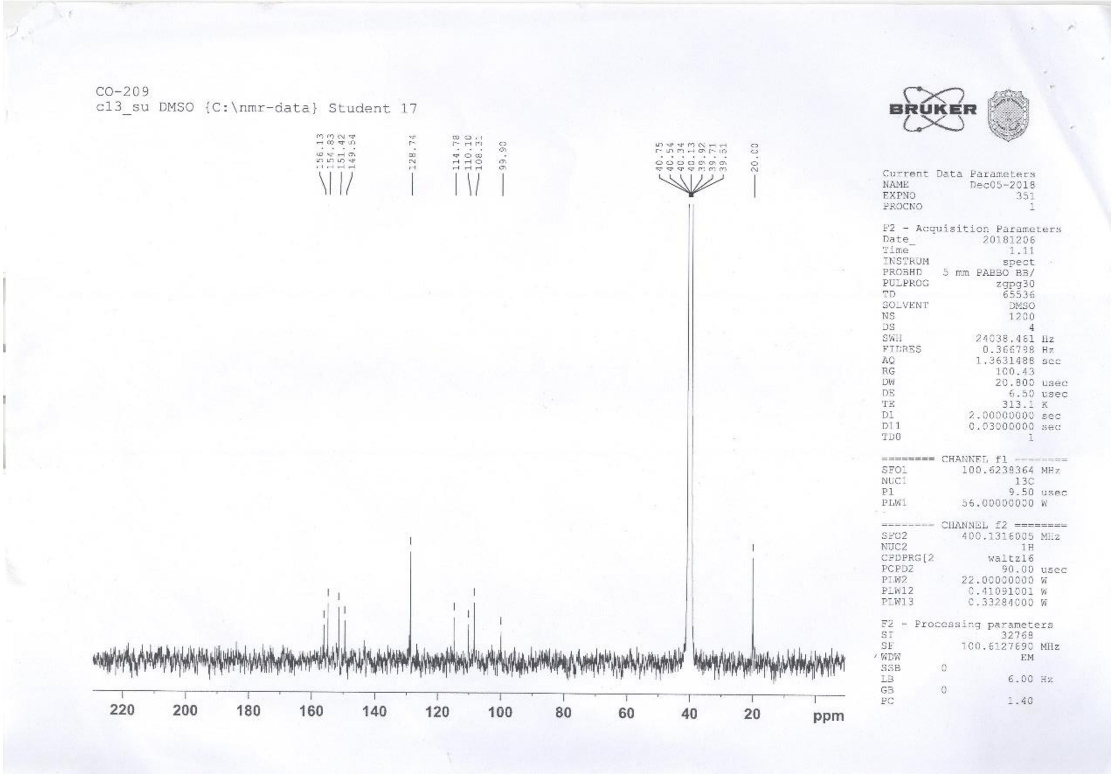

Fig. S4. ^{13}C NMR of Compound (4)

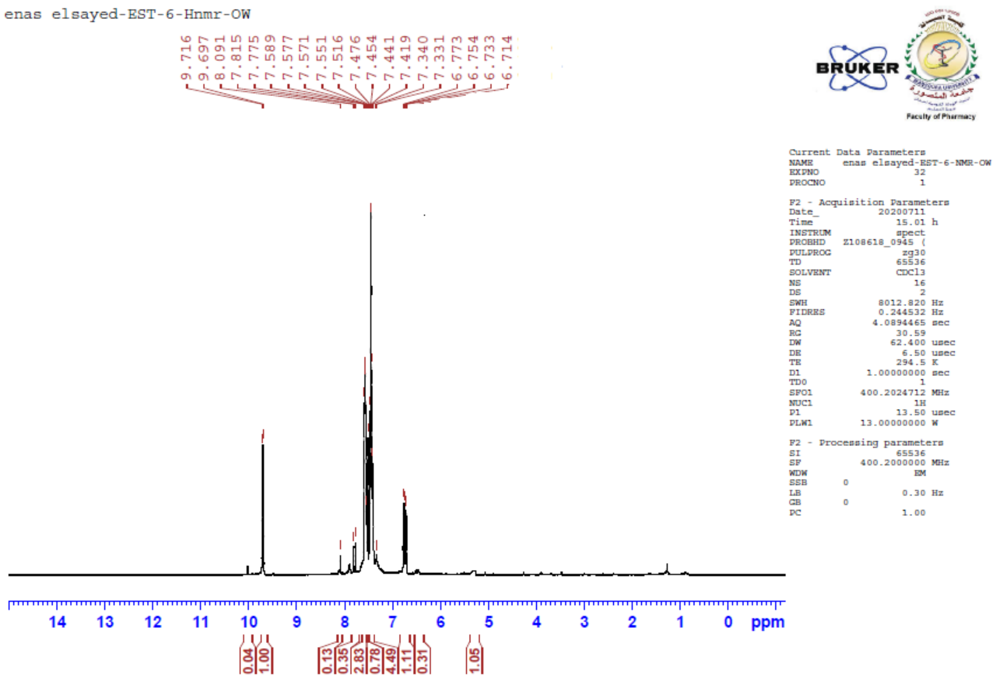

Fig. S5. ^{1}H NMR of Cinnamaldehyde (5)

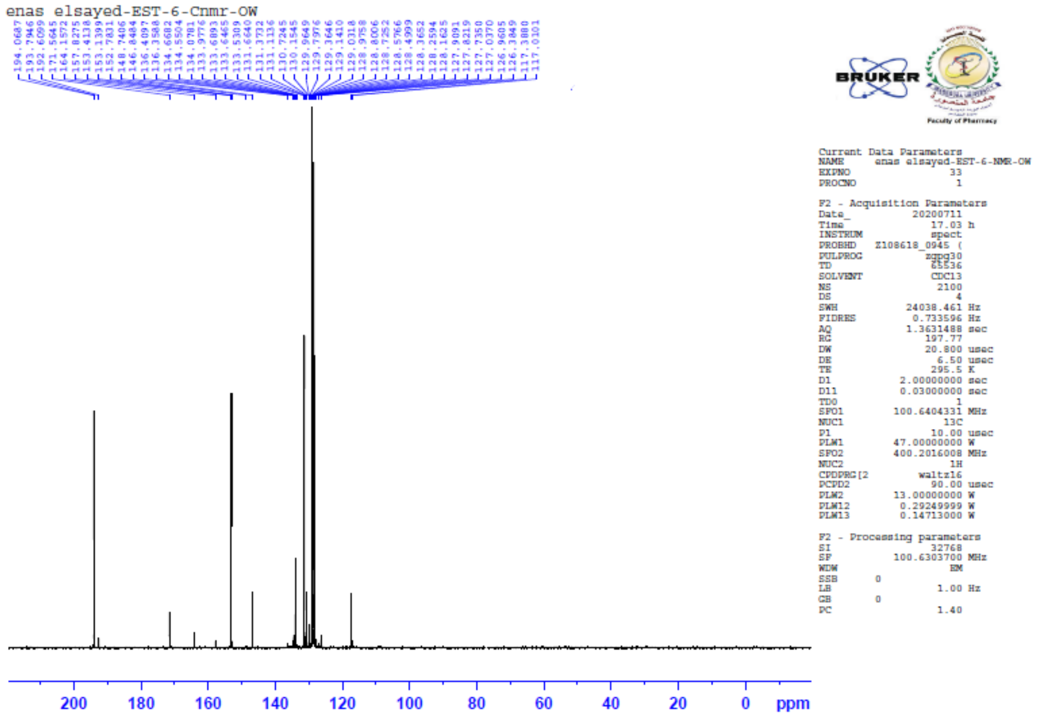

Fig. S6. ^{13}C NMR of Cinnamaldehyde (5)

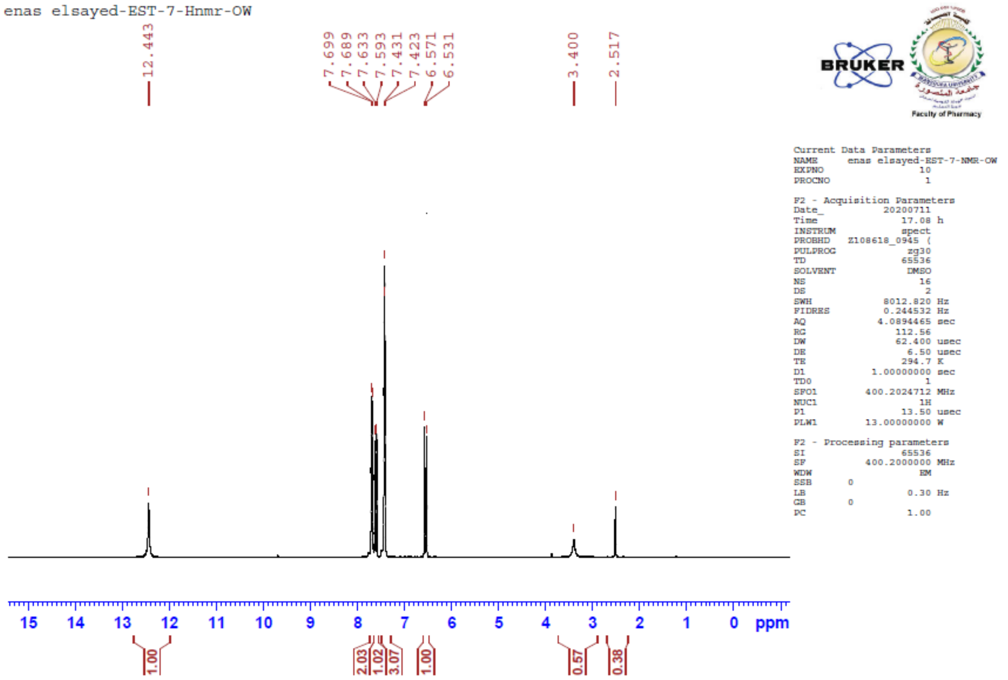

Fig. S7. ^{1}H NMR of Cinnamic Acid (6)

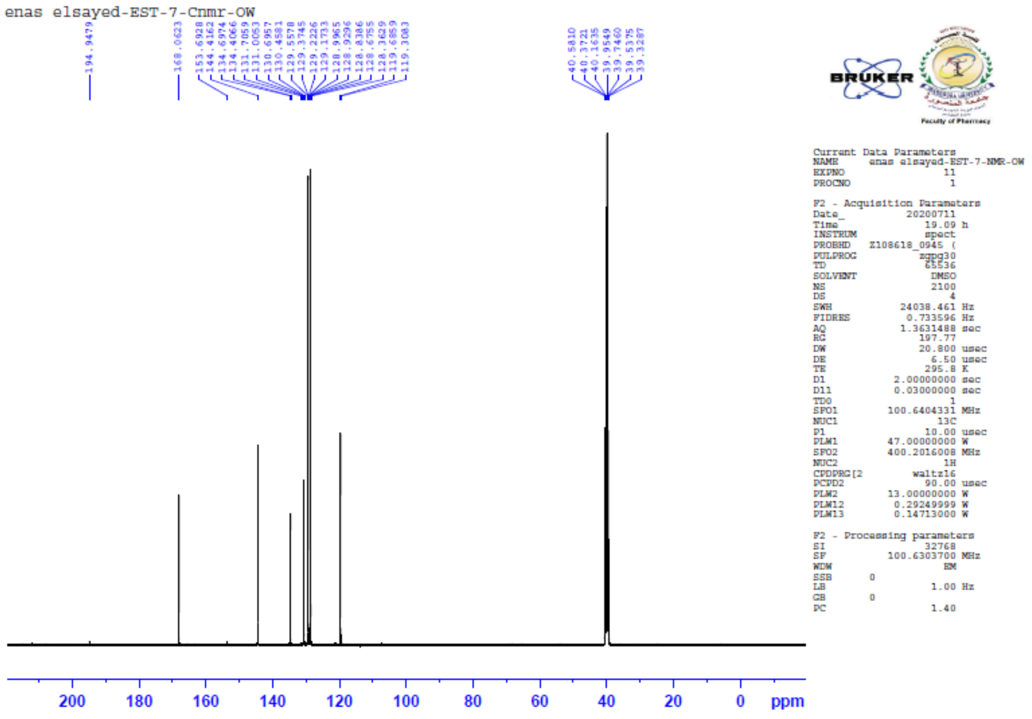

Fig. S8. ^{13}C NMR of Cinnamic Acid (6)

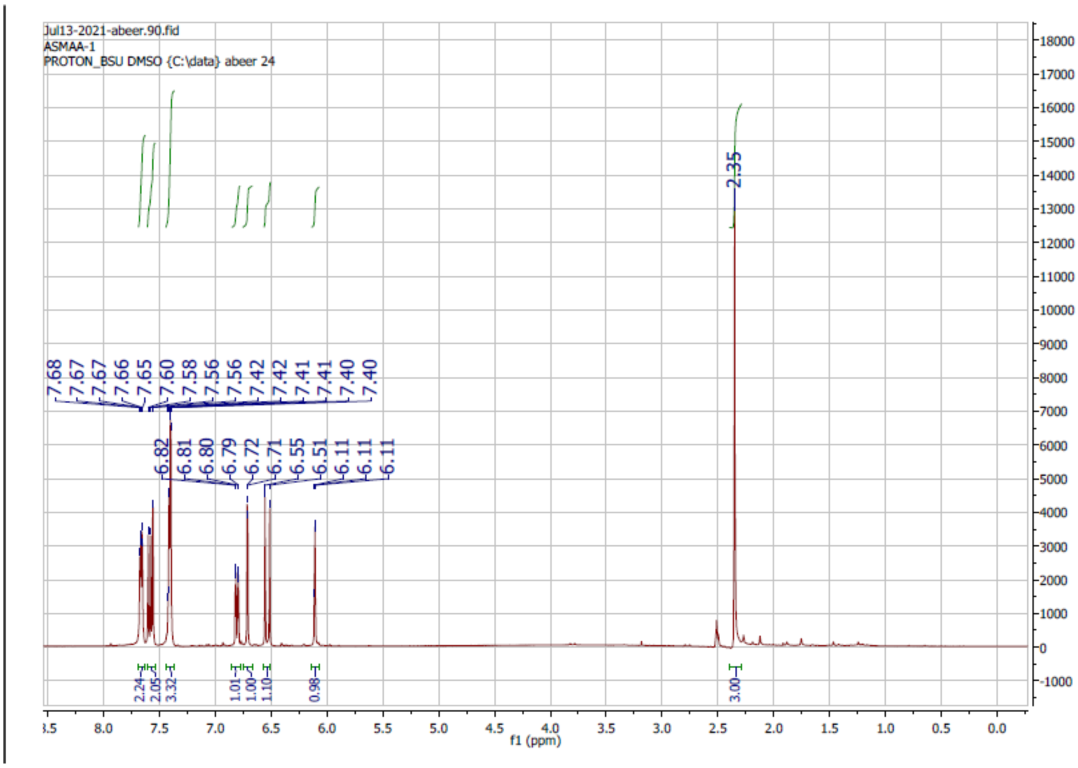

Fig. S9. ^{1}H NMR of Compound (8a)

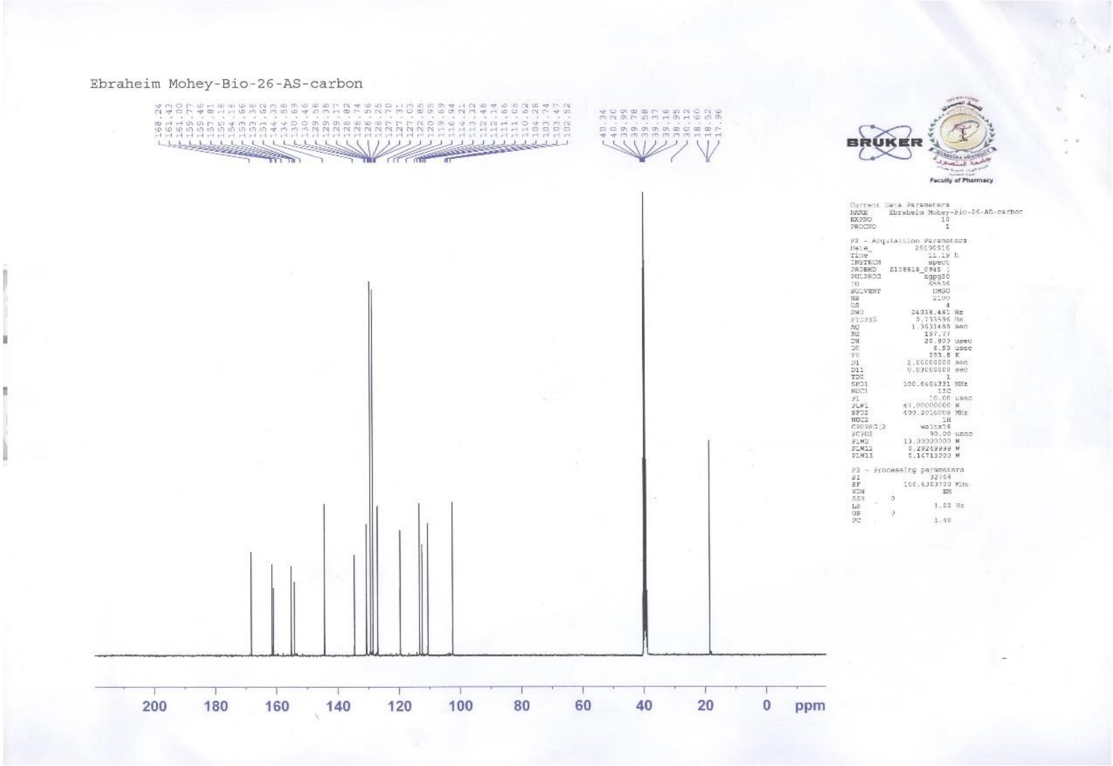

Fig. S10. ^{13}C NMR of Compound (8a)

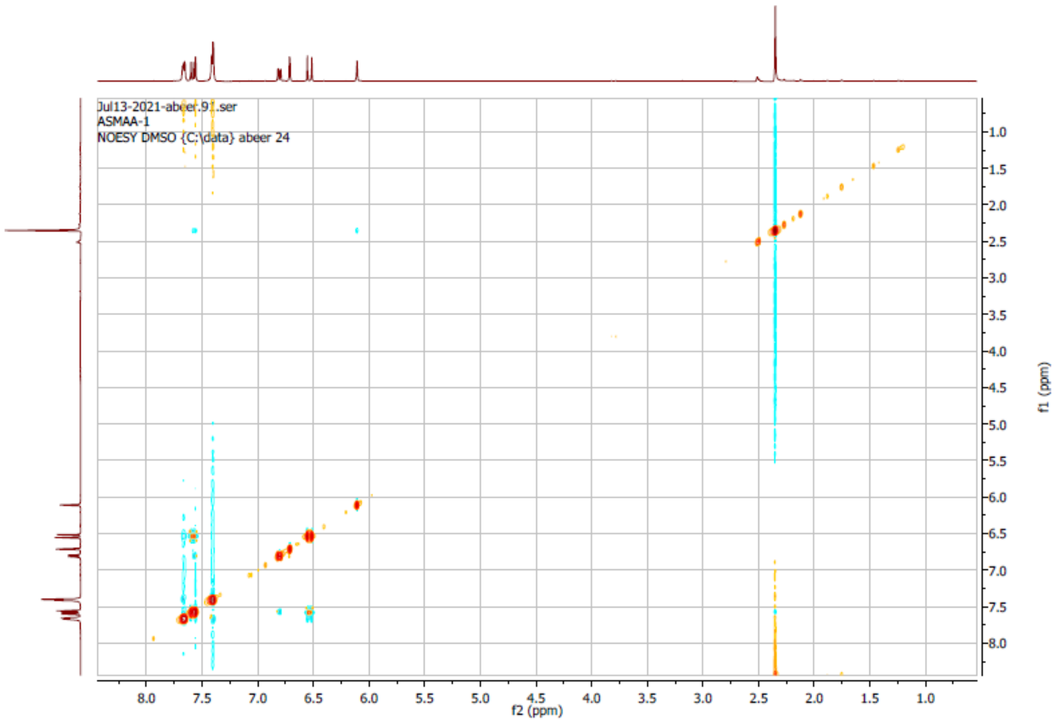

Fig. S11. NOSY of Compound (8a)

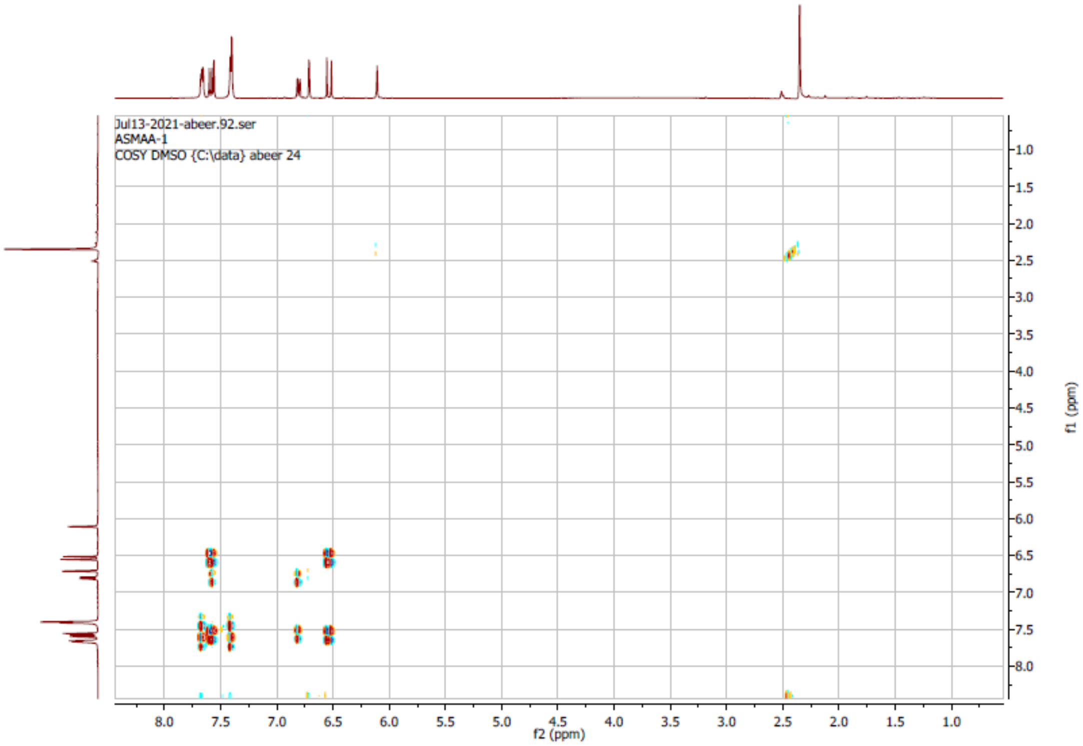

Fig. S12. COSY of Compound (8a)

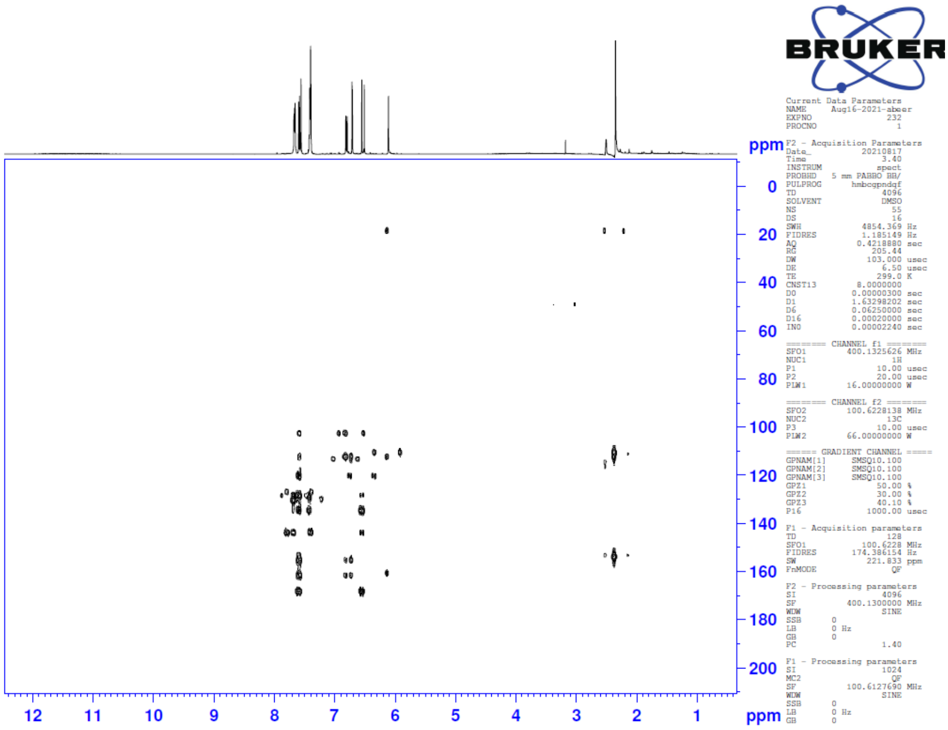

Fig. S13. HMBC of Compound (8a)

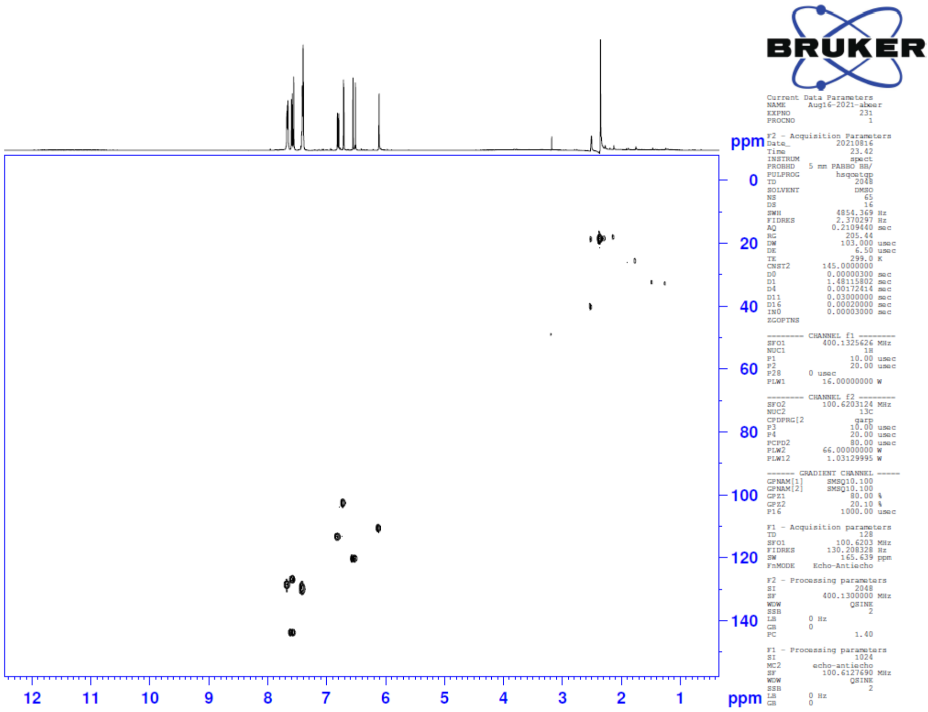

Fig. S14. HSQC of Compound (8a)

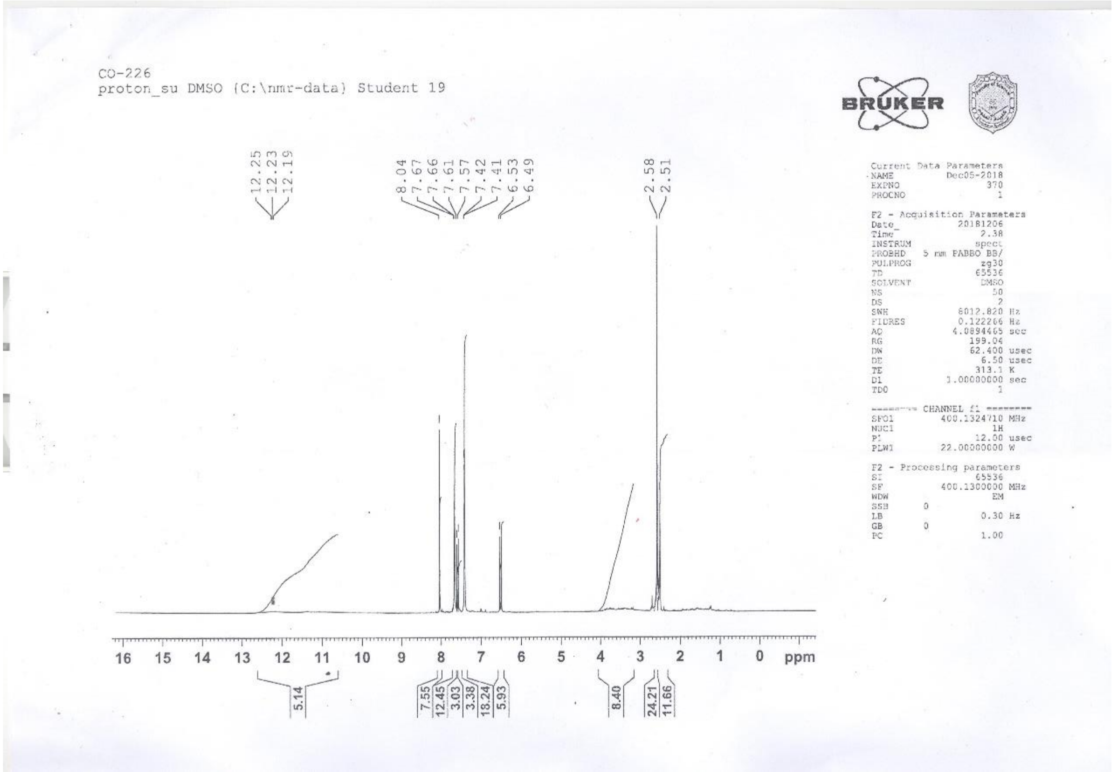

Fig. S15. ^{1}H NMR of compound (8b)

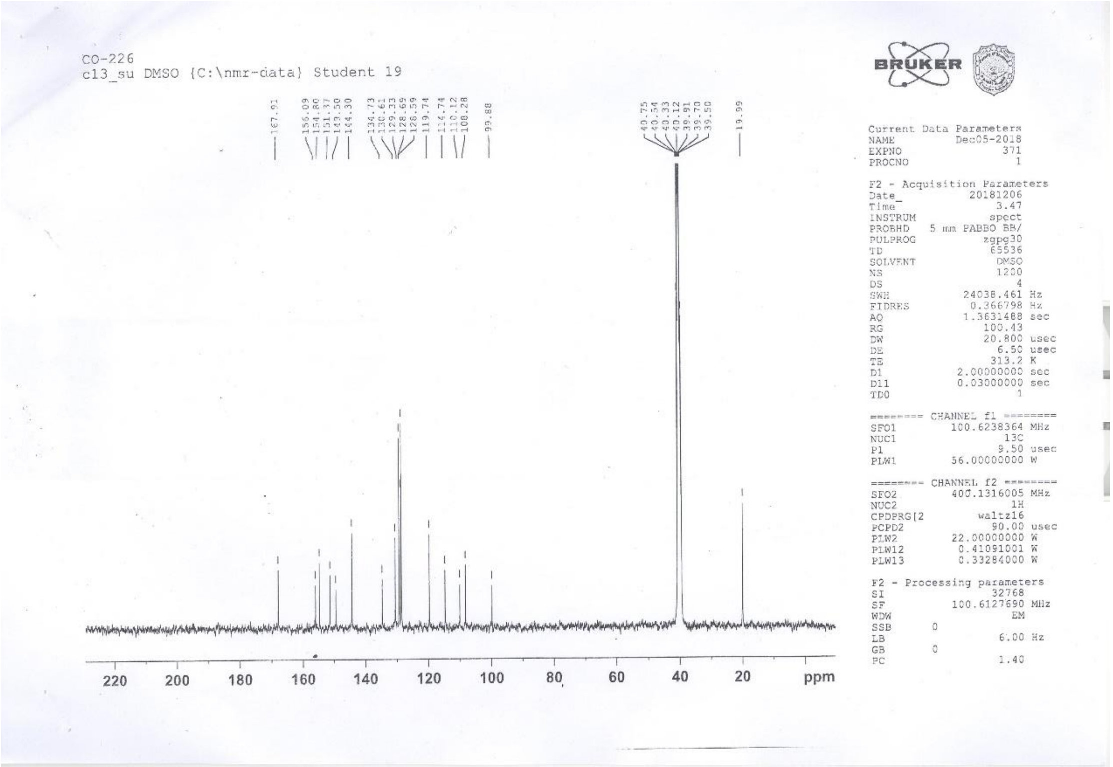

Fig. S16. ^{13}C NMR of compound (8b)

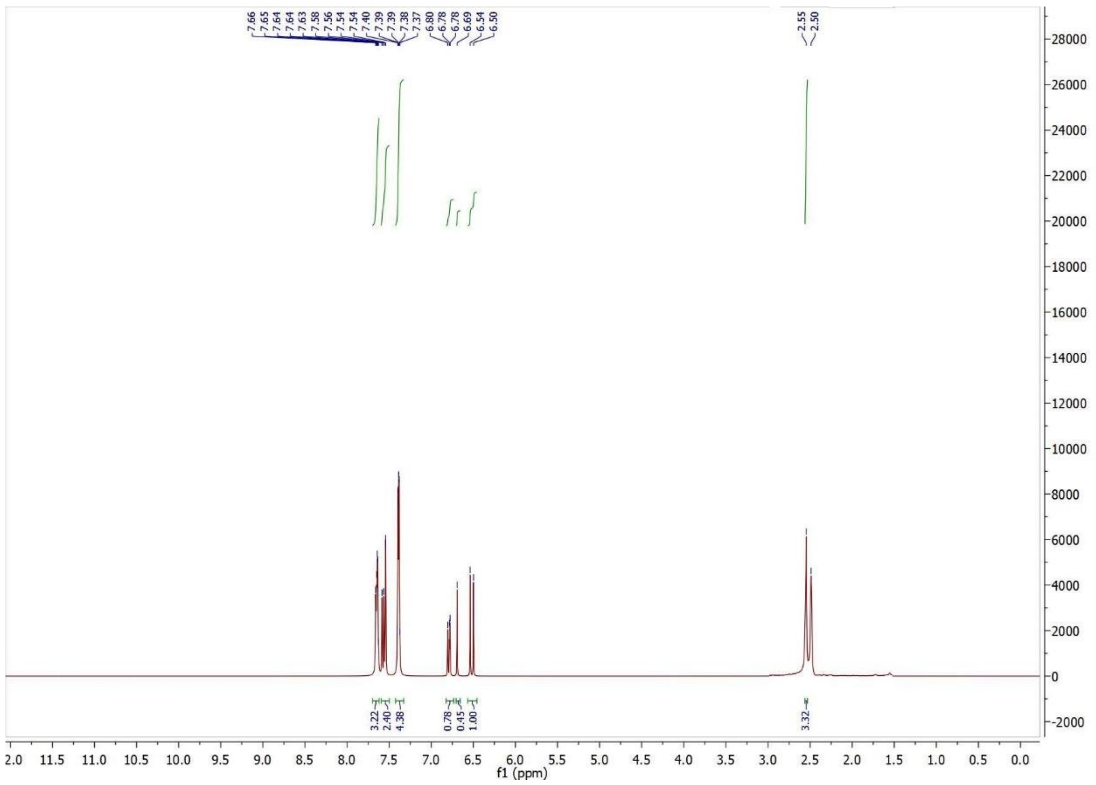

Fig. S17. ^{1}H NMR of compound (8c)

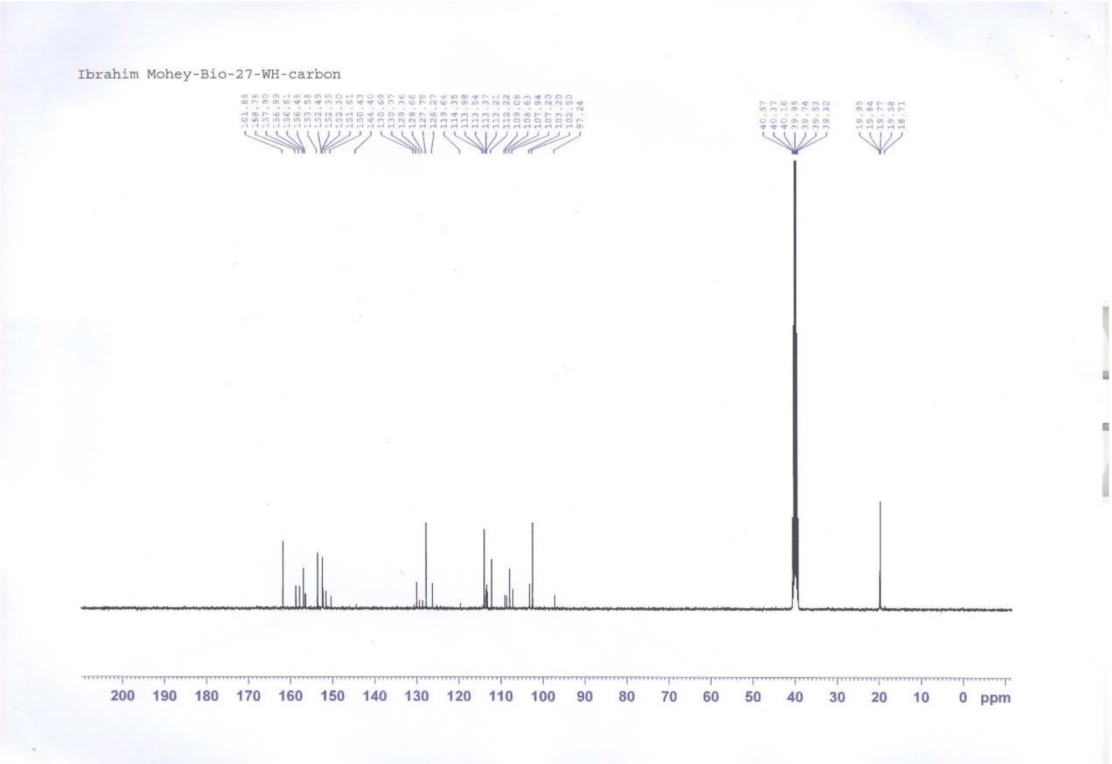

Fig. S18. ^{13}C NMR of compound (8c)

Supplementary materials for detailed methodology

2.2.2.1. Flow cytometric analysis

2.2.2.1.1. *FITC/Annexin-V-FITC/PI Differential Apoptosis/Necrosis Assessment*

Apoptosis and necrosis cell populations are determined using Annexin V-FITC apoptosis detection kit (Abcam Inc., Cambridge Science Park, Cambridge, UK) coupled with 2 fluorescent channels flowcytometry. After treatment with test compounds for 48 h, cells (10^5 cells) are collected by trypsinization and washed twice with ice-cold PBS (pH 7.4). Then, cells are incubated in dark with 0.5 ml of Annexin V-FITC/PI solution for 30 min in dark at room temperature according to manufacturer protocol. After staining, cells are injected via ACEA Novocyte™ flowcytometer (ACEA Biosciences Inc., San Diego, CA, USA) and analyzed for FITC and PI fluorescent signals using FL1 and FL2 signal detector, respectively (λex/em 488/530 nm for FITC and λex/em 535/617 nm for PI). For each sample, 12,000 events are acquired and positive FITC and/or PI cells are quantified by quadrant analysis and calculated using ACEA NovoExpress™ software (ACEA Biosciences Inc.,San Diego, CA, USA).

2.2.2.1.2. *DNA Content-Flow Cytometry Aided Cell Cycle Analysis*

After treatment with test compounds for 48 h, cells (10^5 cells) are collected by trypsinization and washed twice with ice-cold PBS (pH 7.4). Cells are re-suspended in two milliliters of 60% ice-cold ethanol and incubated at 4ºC for 1 h for fixation. Fixed cells are washed twice again with PBS (pH 7.4) and re-suspended in 1 mL of PBS containing 50 µg/mL RNAase A and 10 µg/mL propidium iodide (PI). After 20 min of incubation in dark at 37 C, cells are analyzed for DNA contents using flow cytometry analysis using FL2 (λex/em 535/617 nm) signal detector (ACEA Novocyte™ flowcytometer, ACEA Biosciences Inc., San Diego, CA, USA). For each sample, 12,000 events are acquired. Cell cycle distribution is calculated using ACEA NovoExpress™ software (ACEA Biosciences Inc., San Diego, CA, USA).

2.2.2.1.3. *Caspase- 3/7 green flow cytometry assay*

The ability of compounds **4** and **9** to induce the enzymatic activities of caspase 3/7 in HL60 and HepG2 respectively were assayed using caspase 3/7 green flow cytometry assay method. Briefly, control and the tested compounds treated cells ($2.5x10^5$/mL) were rinsed with ice cold PBS, cell lysates were prepared and subsequently protein

concentration was measured. Lysates were combined with reaction buffer and incubated with Caspase 3/7 Detection Reagent (specific colorimetric substrates) at 37 °C for 6 h. The samples were estimated at λ 488 nm in BD FACS Calibur flow cytometer.

2.2.2.3. Immunoblotting (western plot) for PI3K and AKT

Untreated and treated HL-60 and HepG2 cancer cells were treated with compound **4** (IC_{50}=8.09 µM, 48 h) and compound **9** (IC_{50}=13.14 µM, 48 h), respectively were washed in PBS and lysed in boiling sample buffer (62.5 mM Tris-HCl pH 6.8, 1% SDS, 10% glycerol, and 5% β-mercaptoethanol) for sodium dodecyl sulfate polyacrylamide gel electrophoresis (SDS-PAGE). The lysates were boiled for 5 min with lamellae buffer, and the proteins will be separated by SDS-PAGE and transferred to an Immobilon membrane (Millipore). The antibodies used will be antigen affinity-purified polyclonal sheep IgG anti-human annexin V (R&D Systems). After incubation in 5% non-fat dry milk, Tris-HCL, 0.1% Tween 20 for 1 hr, p-PI3K and p-AKT primary antibodies were added to one of the membranes containing specimen samples and incubated at 4℃ overnight. Appropriate secondary antibodies were incubated for 2 hr at room temperature. After being washed twice n 1 x TBS-T, densitometric analysis of the immunoblots was performed to quantify the amounts of p-PI3K and p-AKT against control sample by total protein normalization by Image analysis software on the ChemiDoc MP imaging system (version 3) produced by Bio-Rad (Hercules, CA). (Tantawy et al 2020)

Table S1. Docked interaction analysis of synthesized ligands screened with the two proteins (PI3K and AKT1).

Ligands	Receptor (Protein)	binding free energy (kcal/mol)	Number of H-bonds	Active site residues	Bonds length (Å)
**3**	PI3K	-9.13	2	Val882<br>Lys833	2.06<br>2.32
	AKT1	-9.60	4	Asp292<br>Ala230<br>Met227<br>Lys179	1.77<br>2.13<br>2.76<br>2.49
**4**	PI3K	-11.80	3	Lys833<br>Asp841<br>Val882	1.98<br>2.60<br>1.98
	AKT1	-13.27	6	Lys179<br>Ala230<br>Thr211	1.98<br>2.45<br>2.65

				Glu228<br>Asp202<br>Asp274	2.90<br>3.00<br>3.03
**6**	PI3K	-9.18	2	Lys833<br>Val882	2.04 -<br>2.52<br>3.17
	AKT1	-10.02	3	Lys179<br>Ala230<br>Met227	2.29<br>1.88<br>3.78
**8a**	PI3K	-12.30	2	Val882<br>Lys833	2.08<br>2.05
	AKT1	-10.06	3	Ala230<br>Lys276<br>Gly157	3.20<br>2.30<br>2.33
**8b**	PI3K	-13.00	4	Asp964<br>Cys869<br>Lys833<br>Val882	3.38<br>3.72<br>2.28<br>2.19
	AKT1	-10.89	6	Lys179<br>Asn279<br>Asp292<br>Gly157<br>Met281<br>Glu228<br>Ala230	2.63<br>3.44<br>2.35<br>3.57<br>2.96<br>3.01<br>3.65
**8c**	PI3K	-12.76	1	Val882	2.05
	AKT1	-10.16	2	Lys179<br>Glu228	1.83<br>2.16
**Staurosporine**	PI3K	-14.07	2	Val882<br>Lys833	1.89<br>1.76
**Spirochromane derivative**	AKT1	-14.90	6	Asp292<br>Glu228<br>Ala230<br>Phe161	1.60<br>2.04<br>2.58<br>2.12<br>2.10<br>2.40
